# Targeting fibroblast growth factor receptors to combat aggressive ependymoma

**DOI:** 10.1007/s00401-021-02327-x

**Published:** 2021-05-27

**Authors:** Daniela Lötsch, Dominik Kirchhofer, Bernhard Englinger, Li Jiang, Konstantin Okonechnikov, Daniel Senfter, Anna Laemmerer, Lisa Gabler, Christine Pirker, Andrew M. Donson, Peter Bannauer, Pia Korbel, Carola N. Jaunecker, Jens-Martin Hübner, Lisa Mayr, Sibylle Madlener, Maria T. Schmook, Gerda Ricken, Kendra Maaß, Michael Grusch, Klaus Holzmann, Bettina Grasl-Kraupp, Sabine Spiegl-Kreinecker, Jennifer Hsu, Christian Dorfer, Karl Rössler, Amedeo A. Azizi, Nicholas K. Foreman, Andreas Peyrl, Christine Haberler, Thomas Czech, Irene Slavc, Mariella G. Filbin, Kristian W. Pajtler, Marcel Kool, Walter Berger, Johannes Gojo

**Affiliations:** 1 Department of Neurosurgery, Medical University of Vienna, Vienna, Austria; 2 Department of Medicine I, Institute of Cancer Research and Comprehensive Cancer Center, Medical University of Vienna, Vienna, Austria; 3 Department of Pediatrics and Adolescent Medicine and Comprehensive Center for Pediatrics, Medical University of Vienna, Vienna, Austria; 4 Department of Pediatric Oncology, Dana-Farber Boston Children’s Cancer and Blood Disorders Center, Boston, MA USA; 5 Broad Institute of Harvard and MIT, Cambridge, USA; 6 Hopp Children’s Cancer Center (KiTZ), Heidelberg, Germany; 7 Division of Pediatric Neurooncology, German Cancer Research Center (DKFZ), German Cancer Consortium (DKTK), Heidelberg, Germany; 8 Morgan Adams Foundation Pediatric Brain Tumor Research Program, Children’s Hospital Colorado, Aurora, CO USA; 9 Department of Pediatrics, University of Colorado Denver, Aurora, CO USA; 10 Department of Neurosurgery, Kepler University Hospital GmbH, Johannes Kepler University, Linz, Austria; 11 Department of Biomedical Imaging and Image-Guided Therapy, Medical University of Vienna, Vienna, Austria; 12 Division of Neuropathology and Neurochemistry, Department of Neurology, Medical University of Vienna, Vienna, Austria; 13 Department of Pediatric Haematology and Oncology, Heidelberg University Hospital, Heidelberg, Germany; 14 Princess Máxima Center for Pediatric Oncology, Utrecht, The Netherlands

**Keywords:** Ependymoma, Brain tumor, FGFR, Small molecule inhibitors, Pediatric cancer

## Abstract

Ependymomas (EPN) are central nervous system tumors comprising both aggressive and more benign molecular subtypes. However, therapy of the high-risk subtypes posterior fossa group A (PF-A) and supratentorial RELA-fusion positive (ST-RELA) is limited to gross total resection and radiotherapy, as effective systemic treatment concepts are still lacking. We have recently described fibroblast growth factor receptors 1 and 3 (*FGFR1/FGFR3*) as oncogenic drivers of EPN. However, the underlying molecular mechanisms and their potential as therapeutic targets have not yet been investigated in detail. Making use of transcriptomic data across 467 EPN tissues, we found that *FGFR1* and *FGFR3* were both widely expressed across all molecular groups. *FGFR3* mRNA levels were enriched in ST-RELA showing the highest expression among EPN as well as other brain tumors. We further identified high expression levels of fibroblast growth factor 1 and 2 (*FGF1*, *FGF2)* across all EPN subtypes while *FGF9* was elevated in ST-EPN. Interrogation of our EPN single-cell RNA-sequencing data revealed that *FGFR3* was further enriched in cycling and progenitor-like cell populations. Corroboratively, we found *FGFR3* to be predominantly expressed in radial glia cells in both mouse embryonal and human brain datasets. Moreover, we detected alternative splicing of the *FGFR1/3-IIIc* variant, which is known to enhance ligand affinity and FGFR signaling. Dominant-negative interruption of FGFR1/3 activation in PF-A and ST-RELA cell models demonstrated inhibition of key oncogenic pathways leading to reduced cell growth and stem cell characteristics. To explore the feasibility of therapeutically targeting FGFR, we tested a panel of FGFR inhibitors in 12 patient-derived EPN cell models revealing sensitivity in the low-micromolar to nano-molar range. Finally, we gain the first clinical evidence for the activity of the FGFR inhibitor nintedanib in the treatment of a patient with recurrent ST-RELA. Together, these preclinical and clinical data suggest FGFR inhibition as a novel and feasible approach to combat aggressive EPN.

**Supplementary Information:**

The online version contains supplementary material available at 10.1007/s00401-021-02327-x.

## Introduction

Ependymomas (EPN) are central nervous system (CNS) tumors which occur across all anatomical compartments and age groups [46]. On the molecular level, nine major groups of EPN with distinct genetic profiles and clinical behavior have been recognized [46]. Posterior fossa (PF) group A (PF-A) and supratentorial (ST) EPNs with gene fusions involving *Zinc Finger Translocation Associated* (*ZFTA, C11orf95*) and *V-Rel Avian Reticuloendotheliosis Viral Oncogene Homolog A* (*RELA*) (ST-RELA) are the predominant groups in the pediatric population and exhibit the highest aggressiveness [46]. Gross total tumor resection and subsequent focal irradiation are considered the mainstay of EPN treatment, whereas the benefit of cytotoxic chemotherapy is controversially discussed [40, 52]. However, complete resection is not possible in approximately one-third of patients [52]. Consequently, novel therapeutic strategies are imperative to reduce the lethality of high-risk EPN. However, the exploration of novel agents against EPN is complicated by the lack of sufficient and appropriate preclinical models. Innovative trials have already explored the potential of novel anti-cancer agents such as the epidermal growth factor receptor (EGFR)-inhibitor lapatinib [10, 18, 20]. Although recent results point towards a benefit of lapatinib treatment in adult EPN patients [20], effective treatments for high-risk EPN in the pediatric population are still missing. Consequently, EPN research has focused on a more profound understanding of the underlying biology aiming at the identification of novel molecularly informed treatment approaches. Genome-wide studies using bulk tissue have explored oncogenic drivers of EPN on the transcriptional and epigenetic level [29, 35, 41, 46]. These studies identified for instance epigenetic dysfunction and aberrant histone modifications caused by *Enhancer of Zeste Homologs Inhibitory Protein (EZHIP)* overexpression or hypoxia as master regulators driving the malignant phenotype of PF-A EPN [29, 35]. Utilizing single-cell transcriptomic approaches we could further define undifferentiated subpopulations and differentiation trajectories within EPN tumor tissues [22, 23]. In these previous studies, we identified fibroblast growth factor receptor 1 and 3 (FGFR1 and FGFR3) as potential therapeutic targets for EPN [11, 23, 35].

The mammalian fibroblast growth factor (FGF) family comprises 18 secreted proteins that interact with four different tyrosine kinase FGF receptors (FGFRs) [14]. Autocrine FGF signaling mediated by FGFRs and/or the respective activating ligands (FGFs) is a frequent event across various cancer types [5]. Indeed, our research group has previously demonstrated oncogenic roles of several FGFR/FGF molecules and their potential as therapeutic targets in glioblastoma, colon carcinoma, lung and hepatocellular cancer [1, 14, 48, 58]. In addition, others and we have also identified alternative splicing of *FGFRs* to increase FGFR signaling and tumor aggressiveness in diverse cancer types [1, 48, 58]. Such splicing events may frequently occur in the immunoglobulin-like domain III of *FGFR1–FGFR3*, generating two major splice variants, referred to as *IIIb* and *IIIc* [65]. The *FGFR-IIIb* and *FGFR-IIIc* isoforms determine the specificity for binding of different FGFs and are related to epithelial-mesenchymal transition [45]. In brain tumors, overexpression, activating mutations and oncogenic fusions of FGFRs have been identified to drive a subset of low- and high-grade gliomas (HGG) [5, 36].

On-target inhibitors of FGFRs are already applied in molecularly guided therapy approaches against several solid tumor types [5, 25, 32, 61], but not yet in EPN. Inspired by comprehensive in silico dataset analyses, we here investigated the oncogenic role of FGFR signaling across molecular EPN groups and the feasibility of FGFR-inhibition to combat this aggressive tumor type.

## Materials and methods

### Human subjects and ethical considerations

The study was approved by the local institutional review board (IRB) of the Medical University of Vienna (EK Nr. 1244/2016). Informed consent to participate in the study was obtained from patients and/or legal representatives of patients treated at the Medical University of Vienna/General Hospital of Vienna.

### Patient material

Tumor tissue was obtained from pediatric patients with intracranial EPNs (WHO grade I, II or III), medulloblastoma (MB) and HGG treated at the Medical University of Vienna between 1992 and 2019. Fresh frozen tissue of four spinal (SP-EPN), three supratentorial subependymomas (ST-SE), one posterior fossa subependymoma (PF-SE), three spinal myxopapillary (SP-MPE), 29 PF-A, five PF-B, ten ST-RELA, and one ST-*Yap1*-fusion-positive ependymoma (ST-YAP1), four MB WNT (MB WNT), seven MB Sonic hedgehog (MB SHH), six MB group 3 (MB G3), three MB group 4 (MB G4), and ten HGG was examined. In addition, 35 formalin-fixed paraffin-embedded (FFPE) samples matched to our frozen EPN tissue samples, comprising 15 PF-A, 5 PF-B, one PF-SE, 4 SP-EPN, three SP-MPE, 5 ST-RELA, and two ST-SE were used for immunohistochemical (IHC) analyses. Molecular subgroups were assigned by methylation array profiling at the DKFZ core unit.

### Cell models and culture conditions

The non-small cell lung cancer (NSCLC) cell line NCI-H1703 and the hepatocellular carcinoma (HCC) cell model Hep3B were obtained from American Type Culture Collection (Manassas, VA, USA) and cultured in RPMI-1640 (Sigma-Aldrich, MO, USA) and EMEM (Sigma) supplemented with 10% fetal calf serum (FCS, Gibco, Thermo Fisher Scientific, MA, USA), respectively. NCI-H1703 served as a positive control for FGFR1 [12], while for FGFR3 Hep3B was used [49, 57]. All primary patient-derived tumor cell models (Table S1) were established from surgical EPN specimens, where patients had given informed consent prior to the operation at the Vienna General Hospital/Medical University of Vienna. Tumor tissue was mechanically dissociated and subsequently grown in RPMI-1640 medium supplemented with 10% FCS for adherent culture, as well as in Neurobasal Medium (NB) (Gibco, Thermo Fisher Scientific) supplemented with 20 ng/ml human FGF2 (PeproTech, NJ, USA), 20 ng/ml human epidermal growth factor (Merck, Darmstadt, Germany), 1% B27, 1% N2 (both, Gibco, Thermo Fisher Scientific, MA, USA) and 2 µM d-glutamin (Merck, Darmstadt, Germany) for spheroid culture. Cells were cultured under standard conditions at 37 °C and 5% CO_2_. All models were periodically tested for mycoplasma contamination. The authentication of established cell models was proven by single-tandem repeat analyses (Microsynth AG, Balgach, Switzerland) and/or methylation array profiling with comparison to matched tumor tissue performed at the DKFZ core facility.

### Cell viability assay

To determine cell viability, cells were seeded in 96 well plates at a density of 4 × 10^3^ cells per well. After a recovery time of 24 h, the cells were exposed to diverse compounds at different drug concentrations in triplicates. The small molecule inhibitors ponatinib, nintedanib, AZD-4547, dovitinib, erdafitinib, avapritinib, and dasatinib were purchased from Selleck Chemicals (Houston, TX, USA). Upon 72 h incubation, cell survival was determined with the commercially available CellTiter-Glo^®^ Luminescent Cell Viability Assay (Promega, Madison, WI, USA) according to manufacturer’s instructions and luminescence signals were measured with the Tecan infinite 200Pro (Zurich, Switzerland). Dose–response curves were generated and anti-cancer activity was expressed as IC_50_ values calculated by GraphPad Prism 8.0.1 (GraphPad Software, La Jolla, CA, USA) using point-to-point function.

### Adenoviral amplification and transgene expression

The expression vector pcHisCtrFGFR, encoding a kinase-truncated, dominant-negative (dn) FGFR1-IIIc (dnFGFR1), was kindly provided by Dr. Francis Kern (Georgetown University Medical Center, Washington, DC, USA) [69]. The truncated FGFR1 fragment was tagged with an enhanced green fluorescent protein (GFP) at the C-terminus by insertion into pEGFP-N3 (Clonetech, Mountain View, CA, USA) to generate a dnFGFR1-IIIc-GFP protein chimera and subcloned into pShuttle-CMV (Strategene, La Jolla, CA, USA). Detailed information describing the creation of the adenoviral expression vector is outlined by Fischer et al. [16]. The expression vector, encoding a dominant-negative, kinase-dead FGFR3-IIIc (dnFGFR3, K508R mutation) was kindly provided by D.J. Donoghue (University of California, San Diego). To achieve a transient expression of dnFGFR3, an adenoviral construct was generated as outlined by Sonvilla et al. [58]. An adenovirus expressing GFP was used as control. Each of the described adenoviral vectors was amplified in HEK293 cells by standard methods. The viral titer was determined with the adeno-X rapid titer kit (Takara Bio Inc., Kusatsu, Japan). Thawing cycles of more than five times of the stored aliquots were strictly avoided to ensure titer consistency.

A multiplicity of infection (moi) of 10 for VBT96, 50 for VBT24,2 or 100 for every other cell model investigated was used to monitor transient gene expression as well as the impact on cell aggressiveness or intracellular signaling. Growth media were exchanged 12 h after infection and proteins were isolated after an incubation time of 72 h.

### Western blotting

Proteins were isolated in 50 mM Tris/HCl (pH 7.6) with 300 mM NaCl and 0.5% Triton X-100, containing protease and phosphatase inhibitors (PMSF, complete, PhosSTOP, Roche, Rotkreuz, Switzerland). Protein concentrations were determined with the Pierce™ BCA Protein Assay Kit (Thermo Fisher Scientific) according to the manufacturer’s instruction. Electrophoresis was performed with 15 µg protein extracts loaded on a polyacrylamide gel and run at a constant 90 V. Subsequently, the proteins were blotted onto polyvinylidene difluoride membranes (PVDF, Thermo Fisher Scientific) and Ponceau protein staining was performed. After blocking, membranes were incubated with primary antibodies (listed in Supplementary Table S2) overnight at 4 °C. β-actin served as a loading control. All antibodies were diluted in 3% bovine serum albumin (BSA, Merck KGaA, Darmstadt, Germany) in Tris-buffered saline-Tween (0.1%) buffer.

### Quantitative real time PCR (qRT-PCR)

RNA was extracted from tumor tissue, using the ReliaPrep™ RNA Miniprep Systems (Promega) or from cell models using TRIzol™ Reagent (Thermo Fisher Scientific). Prior to qRT-PCR analyses, 500 ng RNA was reverse transcribed into cDNA with the RevertAid RT Reverse Transcription Kit (Thermo Fisher Scientific), diluted and mixed with the Maxima Probe/ROX qPCR Master Mix (2×) (Thermo Fisher Scientific). TaqMan probes (Thermo Fisher Scientific) were used to determine expression of *FGFR1* (HS00915137_m1), *FGFR1-IIIb* (HS04260436_m1), *FGFR1-IIIc* (HS00915142_m1), *FGFR3* (HS00179829_m1), *FGFR3-IIIb* (HS1005396_m1), *FGFR3-IIIc* (HS00997397_m1) and *ACTB* (HS99999903_m1). *RelA* expression was analyzed with custom primers (*RelA* fwd CGGGATGGCTTCTATGAGG, *RelA* rev CTCCAGGTCCCGCTTCTT) and compared to the housekeeping gene *RPL41* (*RPL41* fwd CAAGTGGAGGAAGAAGCGA, *RPL41* rev TTACTTGGACCTCTGCCTC) using the GoTaq^®^ qPCR Mastermix (Promega). Reactions were carried out on a CFX Connect Real-Time PCR Detection System (Biorad, California, USA) with standard Taqman or SYBR^®^ Green assay conditions. *ACTB* was used as a housekeeping gene for TaqMan-based qRT-PCR. In every PCR run, a Ct value above 35 was considered negative. In each experiment, the results were normalized to the housekeeping gene for the calculation of ∆Ct values. Detailed quantification is outlined in the respective figure legends.

### siRNA-mediated knock-down of RELA

5 × 10^5^ cells/ml were seeded in 2 ml growth medium into 6 well plates and incubated for 24 h under standard cell culture conditions to recover. On the following day, knock-down was performed using 50 nM RelA-targeting ON-TARGETplus SMARTpool siRNA (Dharmacon, Horizon Discovery Group company, Cambridge, UK) or 50 nM Accell Green non-targeting siRNA (GE Healthcare Little Chalfont, UK). Transfection was performed using Xfect RNA transfection reagent (Takara Bio, Kyoto, Japan) according to company’s recommendations. Total RNA was harvested upon 48 h and proteins upon 72 h incubation under normal cell culture conditions. The respective subsequent experiments (qRT-PCR or Western blot) performed are described above.

### Colony formation assays

To determine the impact on cell survival and cell growth under long-term drug exposure as well as upon blockade of FGFRs with adenoviral constructs (dnFGFR1 and dnFGR3), colony formation assays were performed. In short, 1 × 10^4^ cells were seeded in duplicates in 24 well plates and experiments were started after a recovery time of 24 h. Due to differences in proliferation rates, assays were run 7–14 days. Regarding treatment with inhibitors, every 72 h the media containing the drug was renewed, while upon infection with adenoviral constructs media was changed after 7 days. After incubation, cells were fixed with methanol, stained with crystal violet and photographed with a macro lens on a Nikon D3200 camera (Minato, Tokyo, Japan). For quantification, pictures were binarized using Fiji software [56] and the number of black pixels was subsequently counted. All experiments were performed in duplicates and repeated at least twice.

### Sphere formation and re-attachment/differentiation assays

Formation and growth rate of neurospheres were analyzed in ultra-low attachment 24 well plates (Corning ultra-low attachment multiple well plate size 24, Sigma-Aldrich) with cell counts of 1 × 10^4^ cells per well cultured in NB medium plus supplements. Cells were exposed to inhibitors or to adenoviral constructs immediately after seeding. Inhibitors were renewed after 72 h.

Following six days of incubation, spheres were re-seeded in tissue culture-treated 24 well plates (CytoOne^®^ multiwell plates, Starlab GmbH, Hamburg, Germany) in regular growth media containing 10% FCS and the plasticity of spheres, in this setting attachment and outgrowth, was monitored. During the incubation time, cell vitality was observed daily with a microscope. The plates were incubated for 7–14 days depending on the growth rate and subsequently fixed with methanol and stained with crystal violet. For quantification, the individual wells were photographed with a macro lens (Nikon), pictures were binarized using Fiji software and the number of black pixels was subsequently counted. All experiments were performed in duplicates and repeated at least twice.

### Immunofluorescence staining

5 × 10^5^ cells were seeded in NB medium in ultra-low attachment 6 well plates. Drug (ponatinib 0.5 µM, nintedanib 2 µM or dovitinib 2 µM) was added immediately after seeding and was renewed after 72 h. Following six days of incubation, half of the spheres were re-seeded in the original growth media into tissue culture-treated 6 well plates without further addition of drug. From the second half, cytospins were performed (Epridia™ Cytospin™ 4 Cytocentrifuge, Thermo Scientific) and subsequently spheres slides were fixed for 10 min with ice-cold methanol:acetone (1:1). Re-seeded spheres were allowed to differentiate for 72 h before detachment with TrypLE ™ Express (Gibco). The TrypLE was removed in a washing step with growth media and cytospins were performed. Cells on the slides were fixed with ice-cold methanol:acetone (1:1).

Prior to immunofluorescence staining, spheres or cells were washed three times with PBS, followed by a blocking step of one hour with 20% FCS in PBS. Subsequently, the blocking buffer was removed by washing (PBS) and primary antibody mix solution (1:50 in 2% FCS in PBS) was added. After an incubation of 1.5 h at room temperature, spheres or cells were washed again following incubation with the secondary antibody mix (1:100 in 2% FCS in PBS) for one hour. All antibodies are outlined in Table S2.

After a final washing step with PBS, stained spheres or cells were mounted with DAPI (Vectorshield) and further analyzed using a confocal laser scanning microscope (Zeiss Invert Axio Observer.Z1, Two-channel LSM 700) equipped with LD LCI Plan-Apochromat 25×/0.8 Imm Korr DIC M27 and EC Plan-Neofluar 40×/1.3 Oil DIC M27 objective lenses. Digital images were taken using the Zeiss ZEN software.

### Migration assays

Cell migration was tested by transwell-chamber or wound-healing assays. For the latter, 7.5 × 10^4^ cells were seeded in 48 well plates, and 24 h later the confluent monolayers were wounded using a sterile 200 µl-tip and treated with ponatinib as indicated. To follow cell migration, live-cell microscopy was performed. For quantification, gap-width was measured using Fiji software at the indicated time up to 96 h. Results were calculated as fold change open area relative to the starting gaps. Regarding transwell-chamber assays, 2.5 × 10^5^ cells per well were seeded in Falcon^®^ trans-well culture inserts (Corning, NY, USA) for 24 well plates (8 µm pores) in serum-free growth media. The ability of the cells to migrate through a porous filter was tested after 18 h (VBT211) or 72 h (VBT96 and VBT242) of incubation with 10% serum-containing media as an attractant. Subsequently, migrated cells were fixed, stained with crystal violet and filters were photographed with a macro lens (Nikon). Further quantification was performed as described for sphere formation assays. All experiments were performed in duplicates and repeated at least twice.

### Gene expression arrays

5 × 10^5^ cells were seeded in NB medium in 6 well plates. After 72 h, spheres were treated with ponatinib (1 µM), nintedanib (4 µM) or dovitinib (4 µM). Upon an incubation of 48 h, total RNA was isolated using the RNeasy Mini Kit (Qiagen). Integrity of the RNA samples was checked on an Agilent 2100 Bioanalyzer. mRNA expression arrays were performed with RNAs isolated from two independent experiments.

Whole-genome gene expression arrays (mRNA expression arrays) were performed using 4 × 44 K whole genome oligonucleotide-based gene expression arrays (Agilent) as described in previously [15]. In brief, labelling and hybridization procedures were carried out according to the instructions provided by Agilent using the Quick Amp Labelling Kit and the Two-Color Microarray-Based Gene Expression Analysis Protocol. Scanning was performed on a G2600D Scanner (Agilent). Feature extraction was carried out using the Feature Extraction software (version 11.5.1.1, Agilent).

### Computational analyses of gene expression arrays

Raw expression values were read into the R statistical environment (v.4.0.0), processed, and annotated using the *limma* and *hgug4112a.db* packages [53]. Contrasts of untreated versus treated EPN cell models were calculated using the makeContrasts() function. A ranked list of genes (logFC) served as input for the gene set enrichment analyses (GSEA). GSEA were performed by the *clusterProfiler* package [67] in R using default settings (pvalueCutoff = 0.05, exponent = 1, minGSSize = 15). Results were visualized using the *enrichplot* package by applying the emapplot(), and ridgeplot() functions. Gene sets from the Gene Ontology *Biological Processes (BP) (c5.bp.v7.1), REACTOME (c2.cp.v6.0)*, and *supratentorial EPN (ST)* or *posterior fossa EPN (PF)* [23] were used for GSEAs.

### Single cell RNA sequencing (scRNA-seq) data analysis of EPN datasets

Data for scRNA expression analyses were taken from our previously published studies. Data derived from Gojo et al. [23] were processed and analyzed as published in the respective study. Data previously published within Gillen et al. [22] were derived from the Pediatric Neuro-oncology Cell Atlas (https://www.pneuroonccellatlas.org/) and analyzed with the USCS cell browser (https://cells.ucsc.edu/). 

### Bulk tumor transcriptome analysis

Two independent bulk transcriptome datasets were available for in silico analyses within this study. The Heidelberg dataset comprised mRNA expression profiles analyzed by u133 Affymetrix gene expression array (*n* = 356 EPN, *n* = 657 MB, *n* = 539 pediatric glioblastoma multiforme/GBM, *n* = 506 others) and RNA sequencing (*n* = 25 EPN, *n* = 167 MB, *n* = 30 GBM) from corresponding studies [8, 35, 43]. u133 Affymetrix gene expression data were analyzed with R2 (r2.amc.nl) by plotting the probes FGFR1 226705_at and FGFR3 204379_at. RNA-seq data were processed as previously described [35]. In addition, exon structure reconstruction for detection of alternative splicing was performed from EPN RNA-seq data as well as publicly available human neural stem cell RNA-seq data (GSE76122)[37] with DEXseq tool [3]. The Denver dataset comprised RNA sequencing data of 55 EPN and 59 non-EPN samples.

### scRNA-seq data analysis of developmental and adult brain datasets

Single-cell expression data of the developing mouse brain was generously provided by Sten Linnarsson and obtained from www.mousebrain.org. Data of the adult brain generated with the study of Nowakovski et al. [44] was analyzed utilizing the USCS cell browser (https://cells.ucsc.edu/).

### Analysis of mouse brain atlas

In situ hybridization data was obtained from the Allen Developing Mouse Brain Atlas (Copyright Allen Institute for Brain Science, http://developingmouse.brain-map.org).

### ChIP-seq data analysis of ependymoma tumor tissue

Recently generated [Zheng et al. 70] chromatin immunoprecipitation sequencing (ChIP) data for the activating histone mark H3K27-acetyl (H3K27Ac) and RELA were available to evaluate occupancy at *FGFR1* and *FGFR3* gene loci. ChIP of tumor tissue was performed by Active Motif (Carlsbad, CA, USA) and resulting data was analyzed as previously published [33]. Sequencing reads mapped to hg19 human genome were visualized at the target gene loci utilizing integrative genome viewer (IGV) [54].

### Immunohistochemistry (IHC)

IHC was performed on 3 µm thick sections of FFPE tumor samples on an automate staining platform (Dako Autostainer/Agilent) using a mouse monoclonal anti-FGFR1 (dilution 1:500, #60325-1, ptglabs, Manchester, UK) and rabbit monoclonal anti-FGFR3 (dilution 1:500, #MA5-32620; Invitrogen/Thermo Fisher Scientific) antibody. Stainings were scored using the H-score method which was defined as a continuous variable with a scale ranging from 0 to 300 using the formula: 1 × (percentage of weakly stained cells, 1+) + 2 × (percentage of moderately stained cells, 2+) + 3 × (percentage of strongly stained cells, 3+).

### Statistical analyses

GraphPad Prism 8.0.1 was used to visualize raw data and to analyze statistically significant differences. Data are presented as mean ± standard deviation (SD) as indicated in the figure legends. Appropriate statistical tests were performed as described in the respective figure legends. A *p* value of less than 0.05 was considered to be statistically significant.

## Results

### FGFR1 and FGFR3 expression is enriched in high-risk EPN subtypes

**Fig. 1:**
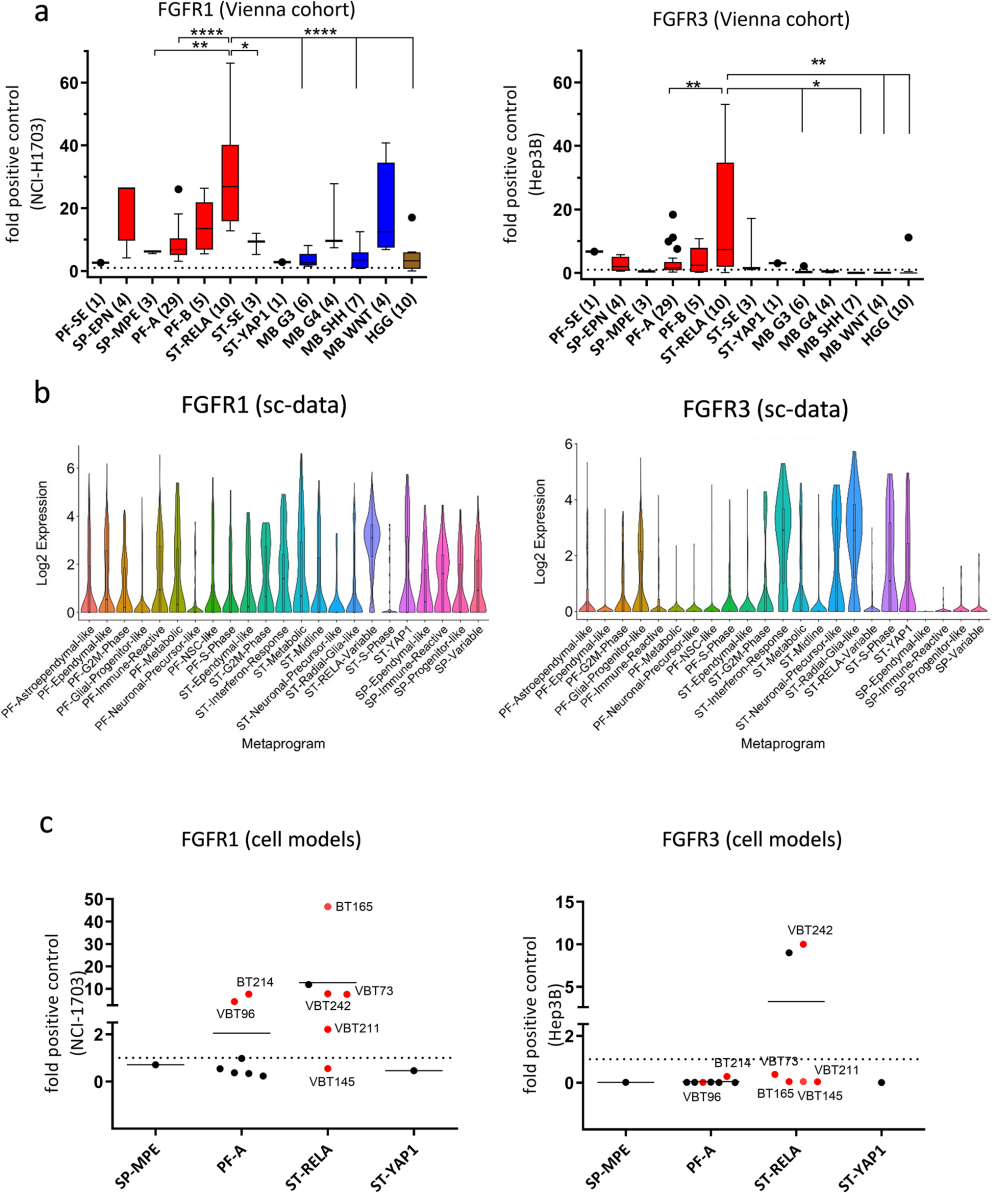
*FGFR1* (left) and *FGFR3* (right) mRNA expression in EPN tissues and cell models. **a** mRNA expression levels were analyzed in 86 surgical samples (Vienna cohort) comprising: posterior fossa (PF) subependymoma (PF-SE), spinal ependymoma WHO grade II (SP-EPN), myxopapillary ependymoma (SP-MPE), posterior fossa group A (PF-A) and B (PF-B), supratentorial (ST) *RelA* fusion-positive (ST-RELA), ST-subependymoma (ST-SE), and ST-*Yap1* fusion-positive (ST-YAP1) ependymomas, medulloblastoma group 3 (MB G3), group 4 (MB G4), sonic hedgehog-activated (MB *SHH*) and wingless-activated (MB WNT) as well as high-grade glioma (HGG). Expression levels were normalized to the housekeeping gene *β-actin* (ΔCT), converted to a linear form using 2^−ΔCT^ and are finally given as fold change (2^−ΔΔCT^) relative to the FGFR-positive controls (NCI-H1703 and Hep3B) shown as dashed line. Numbers in brackets indicate cases analyzed. Significance levels were calculated by one-way ANOVA. ****p < 0.0001, ***p < 0.001, **p < 0.01, *p < 0.05. **b** *FGFR1* and *FGFR3* expression in cellular subpopulations of EPN-derived from scRNA-seq data as published in [23]. *FGFR1* is significantly enriched in the ST-RELA-variable program, *FGFR3* in ST-interferon-response, ST-S-Phase, ST-neuronal-precursor-like, ST-radial-glia-like and in PF-glial-progenitor-like metaprograms. **c** mRNA expression levels in EPN cell models of the indicated subtypes and in the respective FGFR-positive controls (NCI-H1703 and Hep3B) were analyzed by qRT-PCR, normalized to the housekeeping gene *β-actin* (ΔCT) and are finally given as fold change (2^−ΔΔCT^) relative to the FGFR-positive controls (NCI-H1703 and Hep3B). Models used for further investigations are highlighted in red

We and others have previously suggested FGFR1 and FGFR3 as potential oncogenic drivers of EPN [23, 35]. Extent of *FGFR1* and *FGFR3* expression in EPN was analyzed across different molecular groups using three independent cohorts from Vienna (*n* = 56, qRT-PCR; *n* = 35, IHC), Heidelberg (*n* = 356, Affymetrix expression array) and Denver (*n* = 55, RNA sequencing), and further compared to mRNA expression levels in other brain tumor types as well as FGFR-driven tumor models. Interestingly, the mRNA levels of *FGFR1* and *FGFR3* in EPN tissues across all EPN subtypes were at least as high as the levels in the well-described FGFR-driven cancer cell models NCI-H1703 (FGFR1, NSCLC) [12] and Hep3B (FGFR3, HCC) [49, 57] (Fig. 1a left and right panel, respectively). In general, *FGFR1* and *FGFR3* were widely expressed throughout all molecular EPN groups with *FGFR3* being expressed at higher levels as compared to *FGFR1* (Fig. 1a; Supplementary Fig. 1a–d). ST-RELA even exhibited the highest expression of both *FGFR1* and *FGFR3* genes across the entire panel. Remarkably, mean *FGFR3* expression in PF-A, PF-SE, ST-YAP1, and ST-RELA ranked among the highest in comparison to other CNS tumor types (Fig. 1a right panel, Supplementary Fig. 1c, d). IHC staining in the Vienna cohort generally corroborated results from mRNA analyses, again showing expression of both receptors in PF-A, ST-RELA and SP-EPN, whereby expression of FGFR3 was higher as compared to FGFR1 (Supplementary Fig. 1e). Notably, no FGFR1 expression was detected in PF-B although suggested by mRNA expression analyses. Corroborating the IHC analyses, we detected high FGFR3 protein expression in a subset of PF-A and in ST-RELA tissue extracts (Supplementary Fig. 1f). In a next step, we explored co-expression of *FGFR1/3* and FGFs showing that *FGF1* and *FGF2* were highly expressed across all EPN subgroups, whereas *FGF9* was enriched in ST-RELA and ST-YAP1 (Supplementary Fig. 2a, b). Building upon our previous finding that *FGFR3* was enriched in undifferentiated cell populations of ST-RELA [23] we used our two independently generated scRNA-seq datasets, Gojo et al. [23] and Gillen et al. [22], to explore the expression of both *FGFR1/3* and *FGF*s across molecular EPN types, cellular subpopulations and metaprograms. Indeed, within the Gojo et al. dataset [23] *FGFR1* was widely expressed across different cell states and showed the highest expression levels in cells of the ST-RELA-variable program (logFC = 1.53; *p* adjust = 1.69^–83^) (Fig. 1b left panel). *FGFR3* expression was highest in programs related to ST-EPNs (ST-interferon-response, ST-S-Phase—logFC = 2.02; *p* adjust = 9.58^−12^, ST-neuronal-precursor-like—logFC = 1.83; *p* adjust = 4.70^−07^, ST-YAP1—logFC = 1.65; *p* adjust = 3.25^−15^) and glial progenitor-like signatures (ST-radial-glia-like—logFC = 3.30; *p* adjust = 6.67^−118^, PF-glial-progenitor-like—logFC = 0.87; *p* adjust = 1.31^−35^) (Fig. 1b right panel). Analysis of the second dataset published in Gillen et al. [22] confirmed these results, as *FGFR1* was detected across diverse cell states within different molecular EPN groups, whereas *FGFR3* was enriched in ST-RELA, ST-YAP1 and mitotic cells (Supplementary Fig. 3a). With respect to FGFR ligands, we found enrichment of *FGF1* in SP-immune-reactive cells, *FGF2* across diverse PF-A cell types as well as ST-YAP1, and *FGF9* in various ST-RELA and ST-YAP1 cell states (Supplementary Fig. 3b), corroborating our transcriptomic analyses in bulk tissue. Overall, the observed expression pattern of FGF ligands points towards an autocrine stimulation of FGFR1 and FGFR3 in distinct cellular subpopulations within EPN.

We additionally investigated *FGFR1* (Fig. 1c left panel) and *FGFR3* (Fig. 1c, right panel) mRNA levels by qRT-PCR in a set of patient-derived EPN cell models of different molecular groups (*n* = 15). Both receptors were expressed in a subset of PF-A and ST-RELA models, whereby strong co-expression was observed in one ST-RELA model (VBT242). Notably, *FGFR1* and *FGFR3* expression levels were in the range of the well-described FGFR-driven cancer cell models and some of the EPN cell models even exceeded the mRNA levels of the positive controls. Samples highlighted in red in Fig. 1c were used for further investigations. Corroboratively, subsets of PF-A and ST-RELA models, including primo-cell cultures and immortalized cell lines, exhibited FGFR1 and FGFR3 protein expression (Supplementary Fig. 4a). In parallel, we investigated scRNA-seq data generated from EPN tumor models [23]. In VBT242 cells, mRNA expression levels of *FGFR1* and *FGFR3* (Supplementary Fig. 4b) were high, corroborating our qRT-PCR expression analysis (compare Fig. 1c). Interestingly, *FGFR3* was markedly higher in the patient-derived xenograft (PDX) models of BT165 (pink star) and BT214 (yellow star) as compared to the corresponding cell models (pink and yellow hashtag) (Supplementary Fig. 4b). These findings support a direct role of FGFR3 in the tumorigenic capacity of EPN cells harboring a progenitor-like signature.

### FGFR3 is enriched in radial glia cells of embryonic and adult brain

**Fig. 2:**
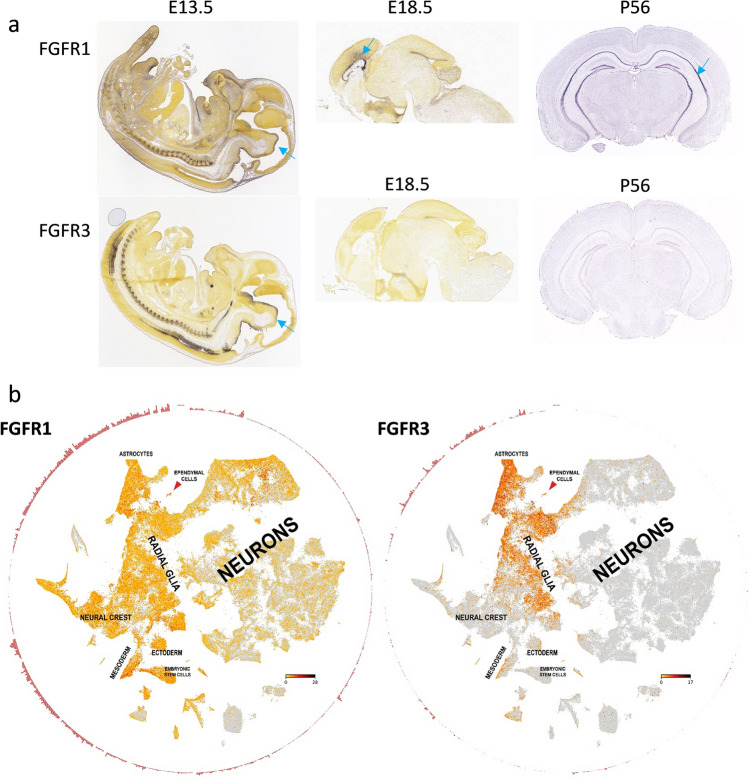
*FGFR* expression of cell populations during brain development. **a** Expression of *FGFR1* and *FGFR3* mRNA in diverse stages of mouse brain development as indicated. In situ hybridization data was derived from the Allen Developing Mouse Brain Atlas (Copyright Allen Institute for Brain Science, http://developingmouse.brain-map.org). Blue arrows indicate the subventricular zone. **b** Single-cell mRNA expression of *FGFR1* and *FGFR3* in diverse cell populations within the developing mouse brain. Data was generously provided by Sten Linnarsson and adapted from www.mousebrain.org. Expression levels are indicated by color (yellow = low; red = intermediate; black = high)

As we recently described parallels between undifferentiated cell populations in EPN and brain development [23], we investigated *FGFR* expression dynamics during mouse embryogenesis utilizing the Allen brain atlas and recently published scRNA-seq data [38]. Indeed, *FGFR1* was expressed in the subventricular zone throughout early and later stages of brain development, whereas *FGFR3* was highest in early stages (e13.5) but was not detectable in the post-natal mouse brain (P56, Fig. 2a). In addition, we analyzed the recently published single-cell atlas of the developing mouse brain and confirmed enrichment of *FGFR3* in radial glia and astrocytes, whereas *FGFR1* was expressed in multiple cell types across brain development (Fig. 2b). Additional analysis of a human cortex data set [44] confirmed the observation in mouse tissue, as *FGFR1* (Supplementary Fig. 5a) was present in both undifferentiated and more mature cell types, whereas *FGFR3* was almost exclusively expressed in radial glia cells (Supplementary Fig. 5b). Taken together, *FGFR3* expression is a characteristic feature of EPN which appears to be derived from its cell of origin, thus corroborating our notion that it is a central mechanism maintaining the stemness phenotype in aggressive EPN.

### Activating FGFR3-IIIc splice variants are enriched in EPN

**Fig. 3:**
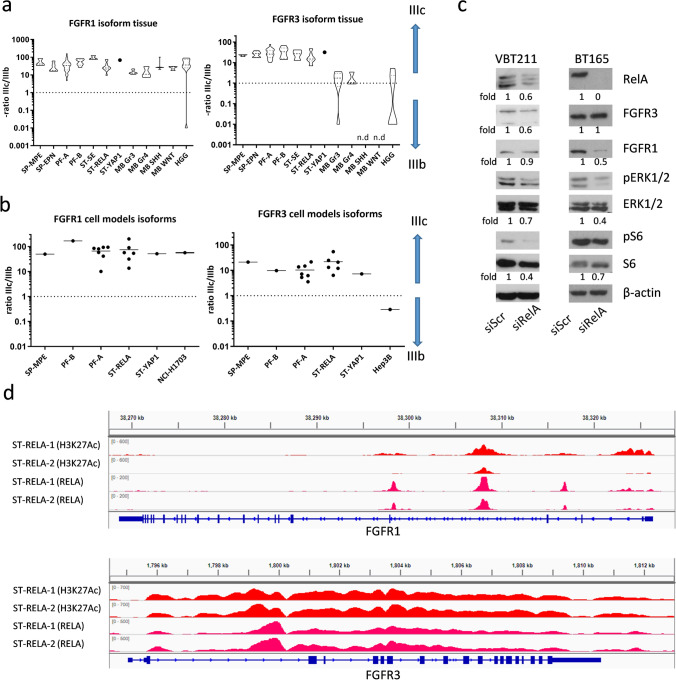
*FGFR1* and *FGFR3* mRNA splice variants in EPN tissue samples and cell models. *FGFR1-IIIb* and -*IIIc* as well as *FGFR3-IIIb* and -*IIIc* mRNA levels in EPN (**a**) tissue samples and (**b**) cell models were determined by qRT-PCR, normalized to the housekeeping gene *β-actin* (ΔCT), converted to a linear form using 2^−ΔCT^ and were finally expressed as ratios IIIc/IIIb. FGFR-positive controls (NCI-H1703 and Hep3B) are included. The cohort comprises myxopapillary ependymoma (SP-MPE), spinal ependymoma WHO grade II (SP-EPN), posterior fossa group A (PF-A) and B (PF-B), supratentorial subependymoma (ST-SE), *RelA* fusion-positive (ST-RELA), and *Yap1* fusion-positive (ST-YAP1) ependymomas, medulloblastoma group 3 (MB G3), group 4 (MB G4), sonic hedgehog-activated (MB SHH), wingless-activated (MB WNT), and high-grade glioma (HGG). **c** Immunoblots depict protein expression and phosphorylation levels 72 h post transfection of the ST-RELA cell models VBT211 and BT165 with siRELA or non-targeting siRNA (siScr). Fold changes of the indicated proteins are given relative to respective siScr controls. **d** Red and pink peaks represent chromatin immunoprecipitation (ChIP) sequencing read coverage for the activating histone mark H3K27-acetyl (H3K27Ac) and RELA, respectively, at *FGFR1* and *FGFR3* gene loci. Sequencing reads of two ST-RELA tissue samples (ST-RELA-1 and ST-RELA-2) were mapped to hg19 human genome and visualized utilizing an integrative genome viewer

Alternative splicing of FGFRs has been shown to regulate receptor stimulation by determining the specific affinity for ligand-receptor interactions [26]. Based on the high expression levels of *FGFR1* and *FGFR3,* we were interested in whether alternative splicing of these receptors is present in EPN. Our analyses demonstrate that *FGFR1-IIIc* isoform is the predominant variant across all pediatric brain tumor types analyzed (Fig. 3a, left panel). In contrast, solely EPNs expressed distinctly higher levels of the *FGFR3-IIIc* variant as compared to the *IIIb* isoform (Fig. 3a, right panel). We further confirmed the presence of the IIIc isoforms in EPN for both receptors in cell models of different molecular groups (Fig. 3b). In addition, data from RNA sequencing analyses in the independent Heidelberg dataset revealed distinctly higher expression levels of the *FGFR1-IIIc* and *FGFR3-IIIc* variants across EPN subtypes (Supplementary Fig. 6a). Corroboratively, we found *FGFR1-IIIc* and *FGFR3-IIIc* to be the predominant variants expressed in human neural stem cells (NSCs) as well as NSC-derived astrocytic cell cultures (Supplementary Fig. 6b).

As the *ZFTA-RELA* fusion protein has been shown to govern transcriptomic patterns in ST-RELA [47], we sought to investigate its influence on *FGFR* expression and alternative splicing. Indeed, recent reports suggest a regulatory function of RELA in alternative splicing events supported by its recruitment to intragenic DNA regions [2]. Accordingly, knock-down of *RELA* in ST-RELA VBT211 significantly reduced expression of the *FGFR3-IIIc* variant as compared to *FGFR3-IIIb,* while in BT165 cells only *FGFR3-IIIb* was slightly diminished*.* Reduction of *RELA* in a non-RELA-driven PF-A (VBT96) cell model had no impact on alternative splicing of *FGFR1* and *FGFR3* (Supplementary Fig. 6c). Next, we investigated the impact of RELA on FGFR protein expression and downstream signaling cascades in two ST-RELA cell models. siRNA-mediated knock-down efficiently reduced levels of the wild-type (wt) and fusion (fus) ZFTA-RELA protein (Fig. 3c, Supplementary Fig. 6d, left panel). In VBT211, FGFR3 expression was distinctly reduced by 40% upon introduction of siRELA, corroborating our qRT-PCR results, while in BT165 FGFR1 was reduced by 50% (Fig. 3c). In both RELA-driven cell models, activation of MAPK and even stronger that of PI3K signaling cascades at the levels of S6 was repressed (Fig. 3c). As expected, knock-down of *RELA* in the PF-A cell line (VBT96) had no impact on FGFR and their downstream signaling pathways (Supplementary Fig. 6d, right panel). Finally, analysis of a recently generated ChIP-seq dataset further demonstrated both transcriptional activation-indicated by H3K27-acetylation-as well as binding of RELA to the *FGFR1* and *FGFR3* loci (Fig. 3d). In summary, our findings suggest a regulatory function of *ZFTA-RELA/RELA-wt* in *FGFR1* and *FGFR3* expression in ST-RELA tumors by transcriptional regulation and, at least in selected cases, also alternative mRNA splicing.

### FGFR blockade impairs cell survival and stemness features in ST-RELA and PF-A EPN cells

**Fig. 4:**
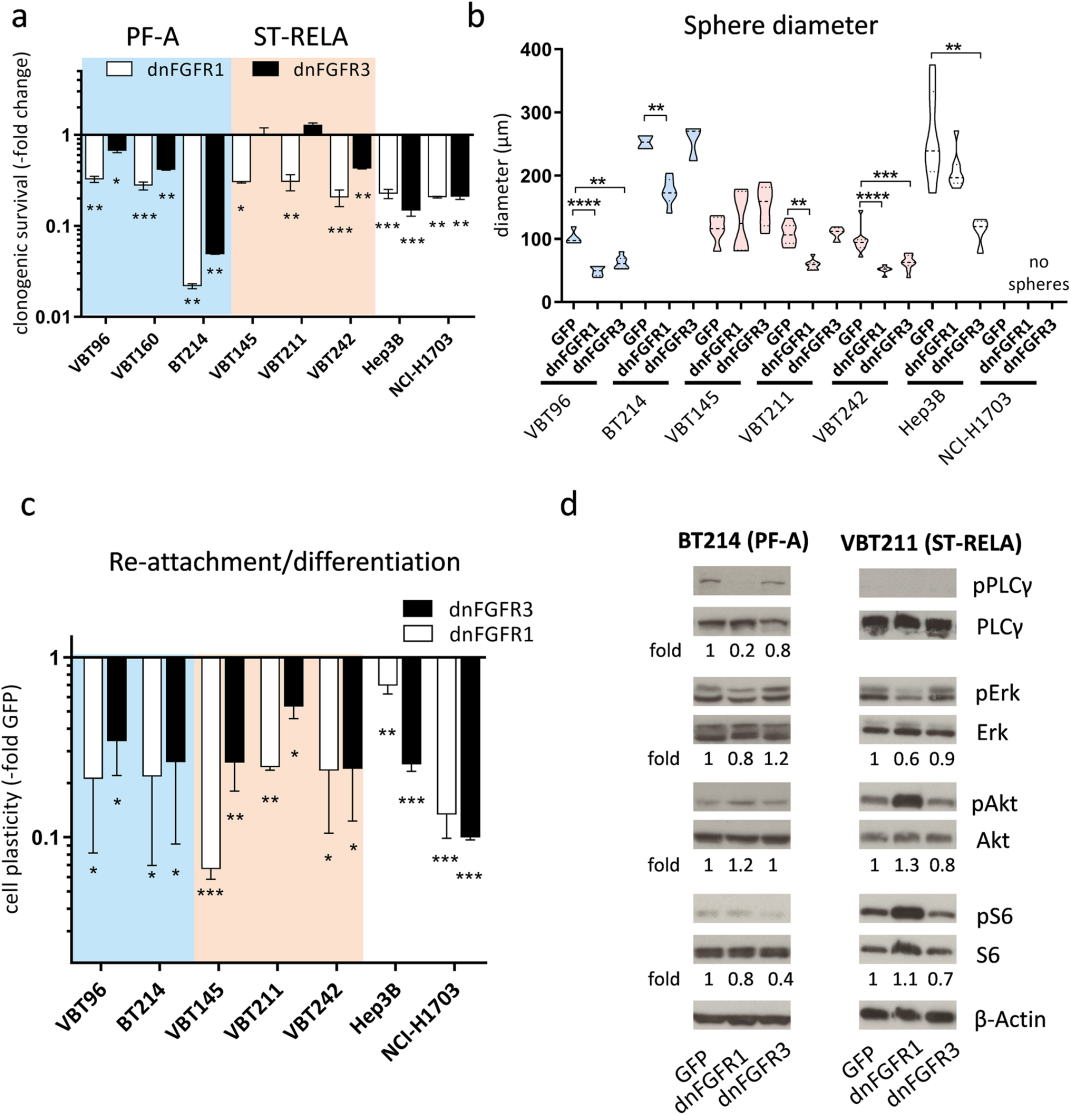
Transduction with dominant-negative (dn) FGFR1 or dnFGFR3 impairs clonogenicity, stem cell capacity and FGFR-downstream signaling cascades. **a** Bar graphs depict fold changes of clonogenic survival upon expression of dnFGFR1 or dnFGFR3 in comparison to GFP-vector controls (set as 1) in the indicated PF-A (blue; *n* = 3) and ST-RELA (pink; *n* = 3) cell models. Cells were seeded at low density, infected with the indicated adenoviral constructs and followed for 14 days. Hep3B and NCI-H1703 served as positive controls. **b** Sphere diameters (in µM) of PF-A (*n* = 2), ST-RELA (*n* = 3) and FGFR-positive control cells expressing either GFP-empty vectors, dnFGFR1 or dnFGFR3 are given. Experiments were performed in duplicates and statistical differences between GFP-controls and dnFGFR1 or dnFGFR3 were determined by one-way ANOVA with Tukey correction for multiple comparison. **c** six days after transduction of the indicated adenoviral constructs (compare **b**), spheres were seeded back in medium containing FCS and the capacity to attach and re-grow was followed. Results are presented as mean ± SD in comparison to respective GFP-vector controls, set as 1. Statistical power was calculated using one-way ANOVA with Tukey correction for multiple comparison. **d** Western blot analyses of the PF-A, BT214 and ST-RELA, VBT211, cell models upon expression of dnFGFR1 and dnFGFR3. Total protein expression and phosphorylation levels of the indicated PLC-γ (PLCγ, pPLCγ), MAPK (ERK, pERK) and PI3K (Akt, pAkt, S6, pS6) pathway mediators are depicted. ß-actin served as a loading control. Fold changes of the indicated proteins are given relative to respective GFP-transduced controls. ****p < 0.0001, ***p < 0.001, **p < 0.01, *p < 0.05

To further elucidate an oncogenic impact of FGFR1 and/or FGFR3 on EPN cell aggressiveness, we transduced ST-RELA and PF-A cell models, as well as the FGFR3-driven HCC Hep3B and the FGFR1-driven NSCLC NCI-H1703 cell lines (all grown under adherent conditions) with a kinase-truncated, dominant-negative FGFR1-IIIc (dnFGFR1) or a kinase-dead FGFR3-IIIc (dnFGFR3) adenoviral construct, comparing effects to a green fluorescent protein (GFP)-expressing control virus. In PF-A cell models, expression of dnFGFR1 or dnFGFR3 either completely blocked (BT214) or significantly reduced (VBT96 and VBT160) clonogenic survival (Fig. 4a, Supplementary Fig. 7a). In ST-RELA cells (VBT145, VBT211, VBT242), blockade of FGFR1 again resulted in decreased clone formation. Expression of dnFGFR3 distinctly impaired clonogenicity in VBT242 cells, representing that model which also showed strongest FGFR3 expression in vitro (Fig. 4a, Supplementary Fig. 7a, compare Fig. 1c).

Following up on our finding that FGFRs are enriched in undifferentiated EPN cell states, we tested how far the introduction of dnFGFR1 and dnFGFR3 affected the stem-cell-like characteristics of EPN cells in comparison to our positive controls, the FGFR3-driven Hep3B and FGFR1-driven NCI-H1703 cell models. Indeed, transduction of PF-A (VBT96 and BT214) and ST-RELA (VBT145, VBT211, and VBT242) cell models with the dnFGFR1 adenoviral construct significantly hampered three-dimensional spheroid growth, indicated by smaller sphere diameters (with the exception of VBT145, Fig. 4b, Supplementary Fig. 7b). We further demonstrate that the expression of kinase-dead FGFR3 distinctly impaired sphere formation in the PF-A (VBT96) as well as in ST-RELA (VBT242) cells. Both models are characterized by the expression of undifferentiated, stem-like EPN signatures which we previously described [23].

To further investigate re-differentiation capacity of the manipulated spheroid cultures, EPN spheres expressing dnFGFR1 or dnFGFR3 were re-grown in a medium containing FCS and the ability of attachment was monitored (Supplementary Fig. 7c). Our investigations in EPN subtypes (PF-A and ST-RELA) reveal that introduction of either dnFGFR1 or dnFGFR3 significantly reduced the ability of attachment and re-growth as two-dimensional monolayer by at least 50%, as compared to GFP-infected cells (Fig. 4c). Summarizing, we demonstrate that blockade of FGFR1 or FGFR3 negatively affects stem cell features indicated by non-adherent sphere formation and re-differentiation capacity of PF-A and ST-RELA EPN cells.

Furthermore, we were interested whether FGFR-mediated signaling cascades, in particular PLCγ, MAPK and PI3K pathways, were altered upon introduction of the kinase-truncated FGFR variants in PF-A (BT214 and VBT96) and in ST-RELA (VBT211 and BT165) cells (Fig. 4d and Supplementary Fig. 7d). In PF-A cells, expression of dnFGFR1 distinctly reduced FGFR phosphorylation (VBT96, Supplementary Fig. 7d) or markedly blocked phospho-PLCγ, a direct substrate of FGFRs (BT214 Fig. 4d), and further downstream reduced PI3K signaling activation at the level of S6. Introduction of dnFGFR3 resulted in comparable reduction of PI3K signaling by down regulation of Akt and/or S6 phosphorylation. In contrast to PF-A, in ST-RELA cells blockade of FGFR1 either resulted in reduced MAPK signaling activation (VBT211), indicated by decreased Erk phosphorylation or down regulation of PI3K signaling (BT165). Presence of the kinase-truncated FGFR3 again reduced Akt or slightly stronger S6 phosphorylation in both ST-RELA cell models. Interestingly, expression of dnFGFR1 increased FGFR3 protein levels in both PF-A and ST-RELA models, suggesting a compensatory regulation between FGFR1 and FGFR3 signaling. In BT165, the upregulation of FGFR3 upon expression of dnFGFR1 was accompanied by induction of FGFR phosphorylation. Taken together, our findings confirm that FGFR1 and FGFR3 hyperactivation in PF-A and ST-RELA cells supports an aggressive stem-cell like EPN phenotype and oncogenic cellular signaling events, thus pointing to an important oncogenic role of these receptors in malignant EPN subtypes.

### FGFR-inhibitors are effective against EPN cell models

**Fig. 5:**
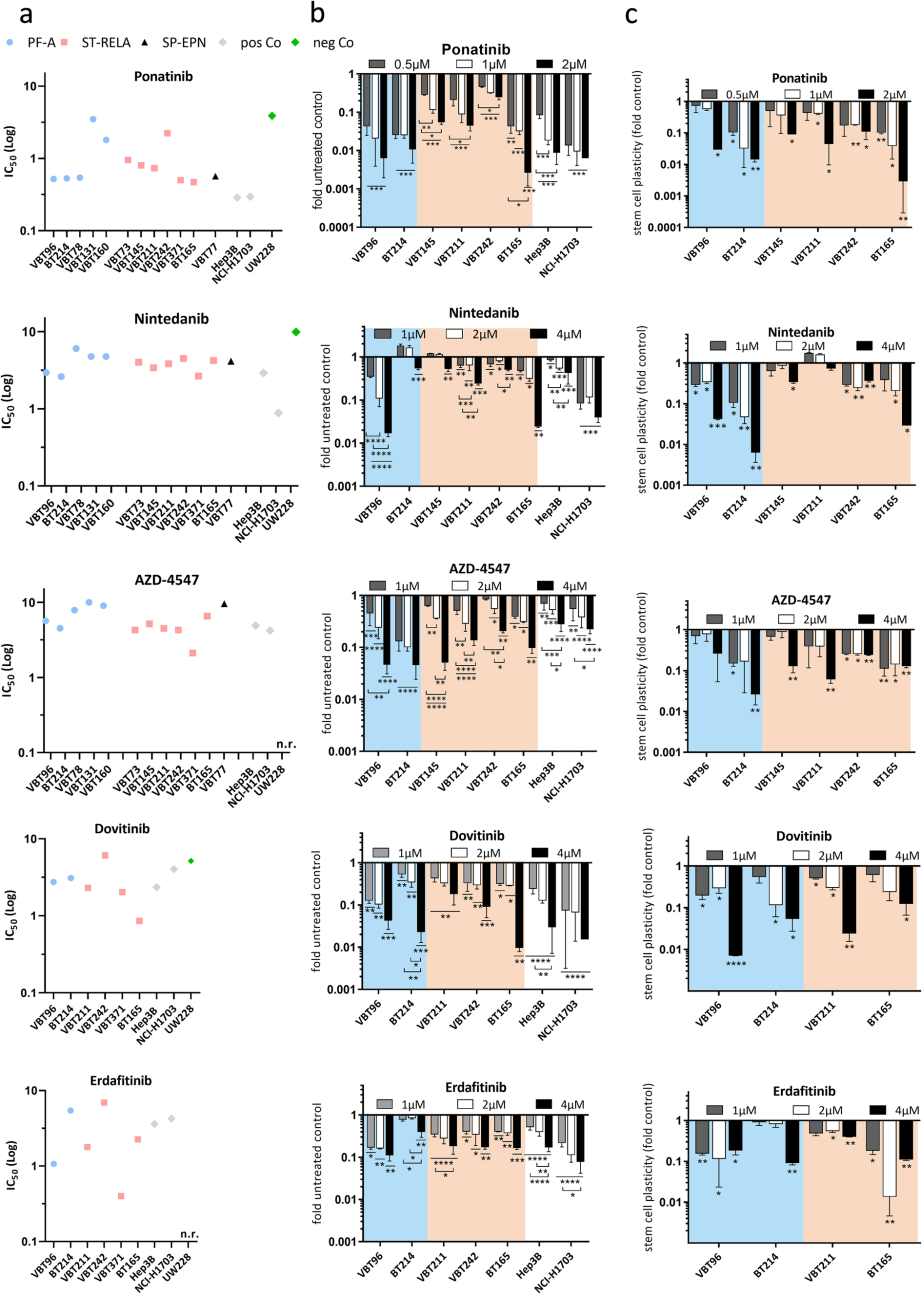
Targeting FGFR with tyrosine kinase inhibitors (FGFRis) impairs survival, clonogenicity and stem cell capacity of EPN cells. **a** Mean IC_50_ values, calculated from three independent experiments, of five different FGFRis (ponatinib, nintedanib, AZD-4547, dovitinib and erdafitinib) are depicted for the indicated cell models of different EPN subtypes in comparison to FGFR-positive (Hep3B, NCI-H1703) and negative (UW228 = MB SHH) controls. **b** Bar graphs depict fold changes of clonogenic survival upon treatment with the indicated FGFRi in comparison to untreated controls (set as 1) in PF-A (blue) and ST-RELA (pink) cell models. Cells were seeded at low density, exposed to the indicated drug concentrations and followed for 14 days. Hep3B and NCI-H1703 served as positive controls. **c** six days after treatment with the indicated inhibitors, spheres were seeded back into medium containing FCS and the capacity to attach and re-grow was followed. Results are presented as mean ± SD in comparison to untreated controls, set as 1. **b**, **c** Every value was evaluated from two independent experiments performed in duplicates and represented as mean ± SD. For **b** and **c** statistical differences between untreated and drug-exposed samples were determined by one-way ANOVA with Tukey correction for multiple comparison. n.r., not reached, ****p < 0.0001, ***p < 0.001, **p < 0.01, *p < 0.05

Considering the high expression levels of *FGFR1* and *FGFR3* across all EPN subtypes as well as the FGFR-driven malignant phenotype of ST-RELA and PF-A tumor cells, we tested the sensitivity of EPN cells towards a panel of small molecule FGFR tyrosine kinase inhibitors (FGFRi). In detail, we selected those that were either already approved in other indications (ponatinib, nintedanib and erdafitinib), harbor the potential to cross the blood–brain barrier (dovitinib), or selectively target FGFRs (AZD-4547). We analyzed the efficacy of the respective FGFRis in a panel of EPN cell models established from surgical specimens of SP-EPN (*n* = 1), PF-A (*n* = 5) and ST-RELA (*n* = 6). Details of these cell models are outlined in Table S1. In general, FGFRis were effective against EPN cells exhibiting IC_50_ values in the nanomolar to low-micromolar range (Table 1 and Fig. 5a). Across the panel, sensitivity was highest against ponatinib (Table 1, mean IC_50_ values outlined in Fig. 5a). Overall, our data indicate that the responsiveness of EPN cells to FGFRis was comparable to FGFR-driven controls and distinctly higher as compared to the negative control UW228 (MB SHH). We further confirmed the efficacy of FGFRis during long-term treatment within clonogenic assays (Fig. 5b and Supplementary Fig. 8). Comparable to the low IC_50_ values in viability assays, ponatinib was the most potent drug, followed by dovitinib, erdafitinib, AZD-4547, and nintedanib. Solely the PF-A model BT214 was more resistant towards nintedanib in the long-term experiment (Fig. 5b). Overall, the investigated FGFRis effectively reduced both cell viability and clonogenic survival in our EPN cell panel.

**Table 1:** Sensitivity of ependymoma cell models towards FGFR inhibitors given as IC_50_ values

	Ponatinib (µM)	Nintedanib (µM)	AZD-4547 (µM)	Erdafitinib (µM)	Dovitinib (µM)
SP-EPN					
VBT77	0.57	4.13	9.58		
PF-A					
VBT78	0.54	6.05	7.88		
VBT131	3.48	4.76	> 10		
VBT96	0.52 ± 0.02	2.95 ± 0.91	5.65 ± 0.02	0.52 ± 0.02	2.75 ± 0.61
VBT160	1.80	4.76	9.03		
BT214	0.53 ± 0.1	2.61 ± 0.75	4.51 ± 2.93	5.44 ± 0.38	3.10 ± 0.90
ST-RELA					
VBT73	0.95 ± 0.37	4.01 ± 1.95	4.27 ± 1.78		
VBT145	0.80 ± 0.34	3.40 ± 0.48	5.16 ± 0.56		
VBT211	0.73 ± 0.45	3.83 ± 1.19	4.50 ± 1.52	1.79 ± 0.21	2.31 ± 0.96
VBT242	2.23 ± 0.25	4.49 ± 1.74	5.67 ± 1.68	6.94 ± 0.74	7.35 ± 0.21
VBT371	0.50 ± 0.05	2.66 ± 0.58	2.11 ± 0.37	0.40 ± 0.07	2.03 ± 1.08
BT165	0.47 ± 0.21	4.23 ± 0.23	6.56 ± 0.83	2.25 ± 0.06	5.16 ± 0.86

Mean IC_50_ values and standard deviations of repeated experiments (if available) for stable cell models are listed

### Treatment with FGFR inhibitors impairs stem-cell-like features of ST-RELA and PF-A EPN cells

As we discovered excellent inhibition of sphere formation and effects on stem cell characteristics upon introduction of dnFGFR1 or dnFGFR3, we investigated whether treatment with FGFRis resulted in comparable effects. Indeed, the mean diameter of EPN spheres was significantly reduced in a dose-dependent manner by FGFRis (quantification in Supplementary Fig. 9 and representative photographs in Supplementary Fig. 10). Again, ponatinib reduced spheroid size at 0.5 µM, whereas the other tested FGFRis distinctly reduced sphere diameter at concentrations of 1 or 2 µM (Supplementary Fig. 9). In addition, effects on EPN cell re-differentiation indicated by adhesion and regrowth of EPN spheres in FCS-supplemented medium were comparable to those observed on the reduction of mean sphere diameter. Interestingly, in contrast to clonogenic survival, in the PF-A VBT96 as well as in two of the investigated ST-RELA cell models (VBT145, VBT211), higher concentrations of FGFRi starting at 1 µM were required to inhibit spheroid attachment to at least 50% (Fig. 5c). With respect to nintedanib treatment, 2 or 4 µM distinctly reduced the capacity of spheres to re-attach and grow in medium supplemented with FCS in all tested EPN cells, except ST-RELA VBT211. Similarly, treatment with AZD-4547, dovitinib, and erdafitinib reduced capacity to re-adhere and proliferate in a dose-dependent manner in every EPN cell model investigated (Fig. 5c). Representative pictures of the attached spheres are outlined in Supplementary Fig. 11a. To further explore the underlying cell biological mechanisms and the impact on stem cell characteristics, we analyzed transcriptomic changes of our previously described [23] intra-ependymal metaprograms upon FGFR-treatment (ponatinib, nintedanib, or dovitinib) of EPN cells. Indeed, we found enrichment of cycling programs (S-phase, G2/M-phase) and undifferentiated programs (NSC-like, Glial progenitor-like, Neuronal Precursor-like) in untreated controls, whereas FGFRi treatment induced expression of more differentiated (Astroependymal-like, Ependymal-like) transcriptomic signatures (Supplementary Fig. 12). To validate the enrichment of differentiation signatures in more detail, we first analyzed protein expression of the astrocytic lineage marker CD44 and the ependymal differentiation factor FOXJ1 following treatment with FGFRi (Supplementary Fig. 11b). Indeed, blockade of FGFR by ponatinib and nintedanib resulted in an increase of FOXJ1 protein expression as well as of CD44 in PF-A cells. We further confirmed differentiation towards ependymal-like cells upon exposure to the FGFRi ponatinib, nintedanib, and dovitinib by performing immunofluorescence staining of the astrocytic differentiation marker CD44, the glial marker GFAP and of the ependymal differentiation factors FOXJ1 and RFX2 in two PF-A (BT214 and VBT96) as well as two ST-RELA (VBT211 and BT165) cell models. Of interest, both ependymal-specific markers, FOXJ1 and RFX2 showed increased expression and partially nuclear localization after treatment and re-attachment of spheres, indicative of differentiation towards an ependymal cell state (Supplementary Fig. 13). While CD44 was slightly increased in treated spheres, under re-differentiation conditions a reduction upon treatment with the inhibitors was observed in cell models of both EPN subtypes (Supplementary Fig. 14, upper panels). Generally, the expression pattern of GFAP was comparable to previously published results of cultured EPN cells [60, 68]. GFAP levels remained unchanged upon exposure to FGFRis and throughout the differentiation process (Supplementary Fig. 14, lower panels). Summarizing, our findings demonstrate that FGFRis effectively impair spheroid growth and induce gene expression patterns related to differentiation in PF-A and ST-RELA cells, corroborating the important biological role of FGFR1 and FGFR3 in these EPN subtypes.

### FGFR inhibition targets the migratory potential of PF-A and ST-RELA EPN cells

As ponatinib impaired clonogenic survival and spheroid growth/cell plasticity in the lowest concentrations, we further investigated the effect of this drug on cell migration. Indeed, ponatinib treatment reduced the migratory potential of PF-A cells (VBT96) already at 0.5 µM in both, filter-migration (Supplementary Fig. 15a) as well as wound-healing assays (Supplementary Fig. 15b). Regarding ST-RELA cell models, VBT145 cells generally lacked migration capacity (data not shown), while VBT242 cells completely lost the ability to move through the pores of the filter (Supplementary Fig. 15a) as well as to close the monolayer wound (Supplementary Fig. 15b) upon ponatinib treatment. Migration of VBT211 cells was reduced in filter migration assays and blocked in wound-healing assays upon exposure to 2 µM ponatinib (Supplementary Fig. 15b). Overall, the inhibitory effects of ponatinib on the migratory potential of EPN cells matched those observed in the investigated controls, NCI-H1703 (Supplementary Fig. 15c, left panel) and Hep3B (Supplementary Fig. 15c, right panel). Corroboratively, in silico analysis showed enrichment of cell migration gene signatures (e.g. focal adhesion, extracellular matrix degradation) in untreated BT214 and BT165 cells as compared to ponatinib treated cells (Supplementary Fig. 15 d) confirming a central role of FGFR signals in ependymoma cell migration.

### FGFR inhibition impedes MAPK and PI3K signaling cascade

**Fig. 6:**
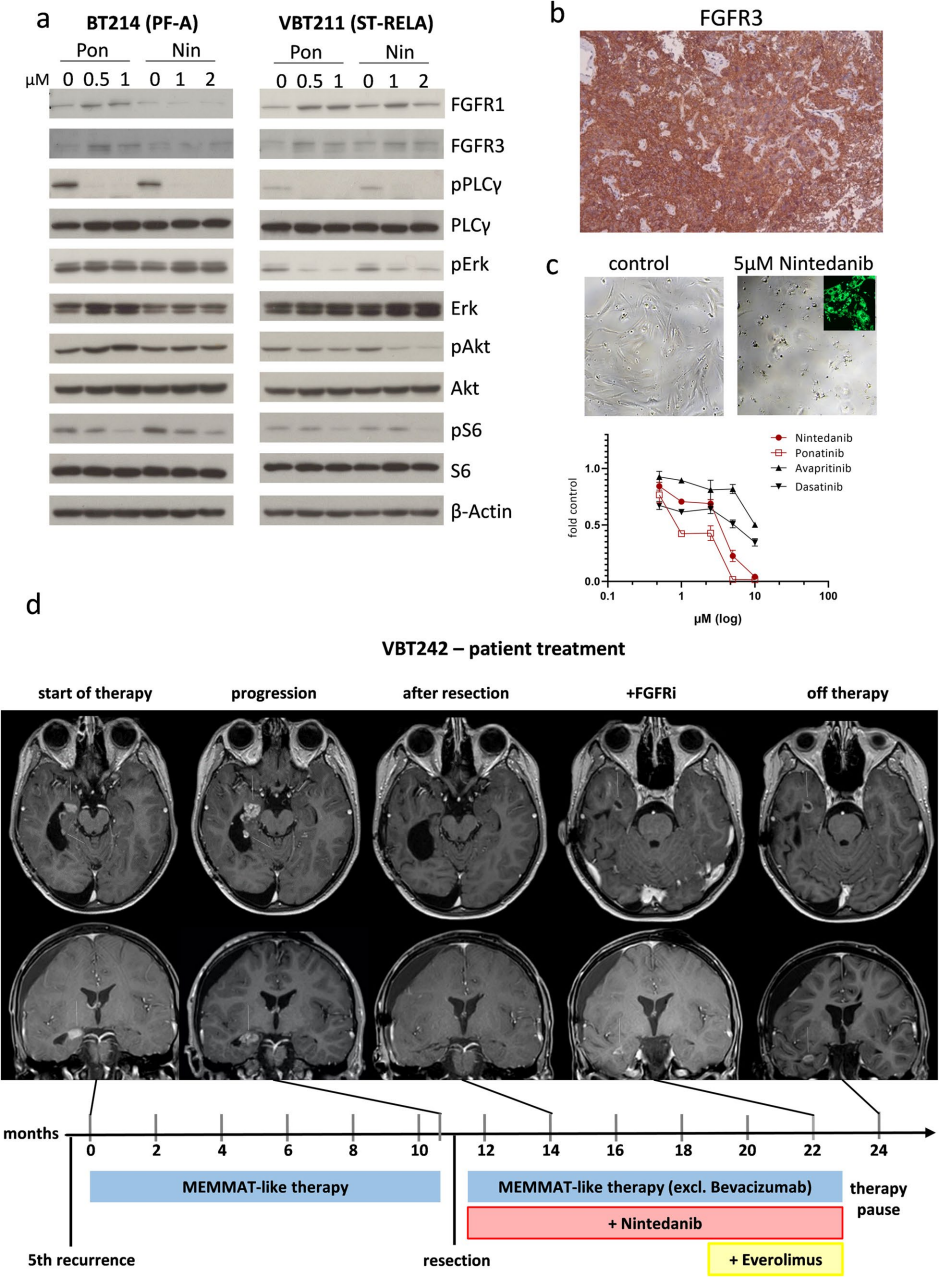
Targeting FGFR inhibits MAPK and PI3K pathway activation, induces differentiation and is applicable in the clinic. **a** Effects of long-term treatment (24 h) with ponatinib (Pon) and nintedanib (Nin) on FGFR1 and FGFR3, on PLCγ, MAPK and PI3K signaling activation (indicated by changes in the phosphorylation status) in PF-A (BT214) and ST-RELA (VBT211) cells was analyzed by Western blot. ß-actin served as a loading control. **b** Immunohistochemical staining of FGFR3 in tissue section of VBT242 at resection prior to FGFRi treatment is depicted. **c** Cell viability of VBT242 upon treatment with the FGFR inhibitor nintedanib and the FGFR/multikinase inhibitor ponatinib, compared to avapritinib and dasatinib targeting PDGFRA and Src, respectively, was determined and is expressed as dose–response curve. Representative pictures at 72 h nintedanib exposure (5 µM) are depicted above. Intracellular accumulation of the drug is verified by the green fluorescence photomicrograph. **d** Course of disease and treatment regimen in a patient suffering from ST-RELA EPN (corresponding to the VBT242 model). Time zero represents the time when the patient was admitted to our center for treatment of the 5th recurrence. The timeline indicates therapeutic interventions and clinical course. MEMMAT-like therapy was carried out as previously published [50] and NCT01356290. Tumor manifestations are indicated with white arrows on axial und coronal contrast-enhanced T1-weighted magnetic resonance images (MRI) at indicated time points. *FGFRi* FGFR-inhibitor

In the next step, we aimed at an investigation of whether treatment with ponatinib and nintedanib also affects receptor downstream signaling cascades. As expected, application of both FGFRis for 24 h resulted in enhanced levels of the two FGFR molecules indicating reduced activation-dependent receptor degradation as described for other receptor tyrosine kinase inhibitors (RTKi) [55]. Accordingly, MAPK, PI3K, and PLCγ FGFR downstream signaling was distinctly inhibited in ST-RELA (VBT211) cells (Fig. 6a), which was comparable to the inhibitory effects seen in the FGFR-driven controls, Hep3B and NCI-H1703 (Supplementary Fig. 16a). In the second ST-RELA model (BT165), ponatinib again efficiently reduced MAPK and PI3K signaling activation. Treatment with nintedanib resulted in decreased PI3K pathway signaling indicated by reduced S6 phosphorylation (Supplementary Fig. 16a). Interestingly, in the PF-A cell model, BT214, both inhibitors completely blocked PLCγ and—to a lesser extent—S6 phosphorylation, whereas no effect was observed on MAPK signaling (Fig. 6a). This indicates selective activation of MAPK-pathway by alternative upstream inducers. To check the role of FGFR as compared to alternative targets of the multi-kinase inhibitor ponatinib, we performed treatment experiments upon stimulation with FGF2 in PF-A (VBT96) and ST-RELA (VBT145 and VBT211) cells (Supplementary Fig. 16b). In line with our hypothesis, treatment with FGF2 induced FGFR phosphorylation across all investigated models and activation of FGFRs and MAPK/PI3K signaling was distinctly inhibited by ponatinib treatment. These data clearly demonstrate that the inhibitory impact of ponatinib treatment on MAPK and PI3K signaling is critically based on FGFR inhibition.

Last, we interrogated genome-wide changes in gene expression patterns induced by FGFRis in EPN cells (Supplementary Fig. 17). These analyses confirm the central impact of FGFRis on regulating cell-cycle programs and cellular proliferation. Interestingly, we further discovered the interaction of FGFR signaling with other cellular processes such as mRNA metabolism, cholesterol/lipid metabolism, PD1 signaling, Notch1 signaling, and ion channels.

### FGFRi treatment of a recurrent ST-RELA case

Based on our preliminary results, we treated a patient with multiple recurrences of a ST-RELA tumor (corresponding to the EPN model VBT242) with FGFRi on an off-label basis. As it was the only FGFRi available for off-label treatment at that time, we chose nintedanib for the treatment of this case. We validated the presence of FGFR3 by IHC staining in the tumor tissue at resection prior to nintedanib application (Fig. 6b). To evaluate the potential contribution of inhibition of other targets such as PDGFRA (nintedanib/ponatinib), and Src (ponatinib), we compared the respective effects to avapritinib (PDGFRA inhibitor) and dasatinib (PDGFRA/Src inhibitor) in the matched cell model VBT242. Clearly, the FGFR-inhibiting compounds exerted superior effects against VBT242 cell survival (Fig. 6c, lower panel). Cell death-inducing efficacy of nintedanib got obvious from photomicrographs of treated VBT242 cells after 72 h exposure, paralleled by efficient cellular uptake of nintedanib detected by fluorescence microscopy as previously published [13] (Fig. 6c, upper panel). Concerning the clinical case, the patient had previously received multiple re-resections (including repeated gross total resections) and repeated focal irradiation as well as systemic chemotherapy. After the 5^th^ recurrence, treatment based on the MEMMAT-regime [50] was initiated, despite of which the tumor showed continuous progression over the following months (Fig. 6d). As a consequence, a re-operation with subtotal resection was performed (Fig. 6d, Supplementary Fig. 18), and MEMMAT based therapy was altered by exchanging bevacizumab for nintedanib. On this regime, the patient showed stable disease for over half a year. At this point, everolimus was added upon further slow tumor progression. This combination, however, was not as well tolerated, leading to medication-induced diarrhea, and the therapy had to be discontinued after one year. As a consequence, the tumor showed fast progression within only 4 weeks after stopping therapy (Fig. 6d). Our comprehensive analysis of this case suggests that targeting FGFR1 and FGFR3 might be an effective treatment of aggressive EPN. Importantly, it has to be considered that nintedanib also targets other kinases, most importantly PDGFRA or VEGFR, and that it was administered within a multimodal treatment. Consequently, this therapeutic strategy will have to be followed up upon in future prospective clinical trials.

## Discussion

FGFRs represent a promising therapeutic target for various cancer types, and FGFR-targeting therapies have already been introduced in selected, non-CNS malignancies [5, 61]. Cross-species and genome-wide epigenomic analyses have identified dysregulation of vesicle trafficking as well as super enhancer activation as drivers of FGFR1- and FGFR3-FGFR1 and FGFR3 activation in EPN [42]. Our systematic dissection of FGF/FGFR signaling in EPN uncovers FGFRs as central mediators of EPN stemness characteristics in highly aggressive subtypes, introduces FGFRis as promising targeted agents for EPN treatment, and gives first evidence for their clinical use against therapy-resistant EPN.

By screening *FGFR1* and *FGFR3* mRNA expression across 467 EPN tissue samples in three independent cohorts, we uncovered that overexpression of these FGFRs is a characteristic feature of EPN across all anatomic locations. Whereas *FGFR3* is almost exclusively expressed in EPN, *FGFR1* levels are also high in MB WNT and ATRTs probably reflecting their derivation from distinct developmental origins [19, 27, 31]. Moreover, we found that *FGFR3* expression is further enriched in malignant supratentorial EPN and ST-RELA in particular. Our data confirm previous analyses based on IHC, already suggesting that increased *FGFR1* and/or *FGFR3* expression in EPN is associated with inferior clinical outcome [34]. In contrast to the respective study, we further included information on the molecular subgroup, thereby showing that FGFRs were particularly expressed in high-risk EPN subtypes. Based on the observation that FGFRs were highest within the ST-RELA group, we further investigated a potential contribution of the ZFTA-RELA fusion protein in inducing expression of *FGFR1* and *FGFR3*. Indeed, siRNA-mediated knock-down led to decreased levels of FGFRs and analyses of ChIP-seq profiles revealed binding of RELA at the respective gene loci. These findings are well in agreement with a recent report which uncovered induction of FGFR-related transcriptomic signatures induced by oncogenic ZFTA-RELA activity [4].

We have recently reported that EPN is driven by aberrant differentiation trajectories and that aggressive subtypes harbor more undifferentiated tumor cells [23]. Making use of our previously published scRNA-seq data, we further found that *FGFR1* expression is present among various subpopulations whereas *FGFR3* is enriched in undifferentiated tumor cells. Corroboratively, we show that *FGFR3* is expressed in the subventricular zone at early developmental stages as well as in radial glia and embryonal astrocytic cells, assumed to be EPN cells of origin [21]. This observation is in accordance with data exploring mouse brain development, where *FGFR3* is expressed by radial glial precursors in the ventricular zone of the embryonic neural tube and is later restricted to differentiated astrocytes [66]. In contrast, *FGFR1* is also expressed at later stages and in more differentiated cell types. These novel findings point towards *FGFR3* as central mediator of the neural-stem cell like properties in EPN inherited from its cellular origin. In this context, it is worth noting that *FGFR3* expression is highest in aggressive subtypes including PF-A and ST-RELA suggesting a central contribution of FGFRs to EPN aggressiveness.

Apart from overall expression, FGFR1 and FGFR3 activity is regulated by alternative splicing, resulting in different ligand-receptor affinity [26, 45]. We have previously described FGFR1 or FGFR3 high-affinity *III*c variants as mediators of cancer cell aggressiveness in lung and colorectal cancer [16, 58]. In EPN, we found that both receptors were predominantly present in the more active *IIIc* variant, further supporting aberrant activation of FGFR signaling. Whereas *FGFR1-IIIc* was also the predominant form in other CNS tumor types as well as in our FGFR-driven control cell models, only EPN cells exhibited extensive expression of *FGFR3-IIIc*. Interestingly, in silico analysis of human NSCs confirmed alternative splicing in radial glia cells and derived astrocytic cell cultures. These findings are well in line with previous reports underlining the role of *FGFR-IIIc* variants in brain development [17, 62, 64]. We further investigated potential activating factors of alternative splicing and could define RELA signaling as a mechanism supporting the preferential expression of the *FGFR-IIIc* isoform. Recently, a regulatory function of RELA in alternative splicing events has been described, which may also play a role in ST-RELA. In detail, RELA has been shown to bind to GC-rich exons, thus recruiting the splicing factor DDX17, which regulates splicing via its RNA helicase activity [2]. Regarding PF-A, *FGFR3-IIIc* is activated via WNT signaling in colorectal cancer [58], and we have previously described potential activation of this pathway via *LGR5* expression in undifferentiated PF-A cells [23]. Consequently, WNT signaling may also play a role in FGFR activation within EPN.

Next, we confirmed the oncogenic role of FGFR1 and FGFR3 in EPN by dominant-negative inhibition of FGFR signaling using adenoviral constructs, which inhibited cell growth, neurosphere formation, and stem cell characteristics of patient-derived EPN cell models. Notably, these effects were comparable to those observed in other well-described FGFR-driven tumor types such as HCC, lung and colon cancer [14, 48, 59]. Moreover, blockade of FGFR1 and FGFR3 was sufficient to inhibit central oncogenic pathways including PLCγ, MAPK and PI3K, confirming dependency on autocrine FGFR activation. In detail, expression of dnFGFR1 fully inhibited phosphorylation of PLCγ in PF-A cells, highlighting the role of FGFR1 in this EPN subtype. Accordingly, selective PLCγ activation has been described via binding of distinct SH2 domains to phosphorylated tyrosine residues within the FGFR1 kinase domain [6, 28].

To evaluate FGFRs as clinically actionable targets, we performed drug testing including a broad range of FGFRis and a panel of 12 EPN cell models. These analyses demonstrated the overall remarkable sensitivity of EPN towards various FGFRis with IC_50_s in the low-micromolar to nanomolar range. In general, the IC_50_ values of FGFRis were comparable to FGFR-driven control models [14] but also to other RTK-driven pediatric brain cancer cell models including NTRK fusion-driven HGG cell models towards NTRK-inhibitors [9, 39]. High sensitivity was observed not only for the multi-RTKi ponatinib, but also the more FGFR-specific AZD-4547 inhibitor, proving FGFRs as central therapeutic targets in EPN cells. Corroboratively, AZD-4547 was active against an EPN PDX model [35] and the experimental pan-FGFRi BGJ398 inhibited EPN cells derived from animal models [42]. Besides cell viability, in our hands FGFRis demonstrated also profound effects on neurosphere forming capacity, cell growth, differentiation patterns and cell migration. In accordance with the adenoviral intervention, we proved distinct inhibition of major oncogenic signaling pathways, including MAPK, Akt, and PLCγ, in both PF-A and ST-RELA models upon pharmacological FGFR inhibition. Interestingly, FGFRi treatment resulted in induction of astroependymal-like signatures as well as FOXJ1 and RFX2, both factors associated with a more differentiated ependymal-like cell state in brain development and ependymoma [23, 30]. Importantly, we have previously shown that these transcriptomic signatures are associated with a more favorable clinical outcome [23]. Consequently, our results suggest that FGFRis induce maturation in aggressive ependymomas, a therapeutic concept currently considered of high potential in pediatric cancers [7]. Last, we found that FGFRis had a central impact on multiple cell biological processes including lipid and RNA metabolism as well as Notch1 or PD-1 signaling, some of which have previously been described for being deregulated or hyperactivated in ependymoma [42, 51, 63].

As already mentioned, currently no effective systemic treatment strategies for EPN are available. Building upon our preclinical data, the ST-RELA case corresponding to the VBT242 cell model was therefore treated at 5th recurrence with nintedanib, the only FGFR inhibitor available for off-label use at that time. In combination with a MEMMAT backbone [50] treatment (omitting bevacizumab), addition of nintedanib resulted in stable disease for 11 months following rapid tumor progression after therapy interruption and subsequent MEMMAT treatment without the addition of FGFRi. Considering that nintedanib is substrate to ABCB1-mediated efflux [14] and has only limited blood–brain-barrier penetration [24], alternative highly brain-penetrant FGFRis might even exert more pronounced anti-EPN effects. Together this suggests the feasibility of FGFR inhibition-based EPN therapy in a clinical setting although it has to be considered that nintedanib was administered within a multimodal treatment approach. Still, taking into consideration the matched in vitro data of the respective case, our data support further investigation of FGFRis against ependymoma in future clinical trials.

In summary, we show that oncogenic activation of FGFR signaling widely contributes to the malignant phenotype of aggressive EPN. Moreover, we find that higher *FGFR3* expression is specific for EPN, well in accordance with its developmental origin. We further prove that preclinical EPN models are susceptible towards FGFRis and provide first practical evidence for their potential clinical use in the treatment of EPN patients. Based on our study, clinical trials investigating the efficacy of FGFRis to combat aggressive EPN are warranted to further pursue this promising novel therapeutic strategy.

## Supplementary Information

Below is the link to the electronic supplementary material.

Supplementary file1 (PDF 20795 KB)

Supplementary file2 (XLSX 413 KB)
