# Supplementary material for: Targeting fibroblast growth factor receptors to combat aggressive ependymoma
Source: Acta Neuropathol. 2021 May 27;142(2):339–60. doi: 10.1007/s00401-021-02327-x (PMC8270873; doi:10.1007/s00401-021-02327-x)
Supplement: Supplementary file 1 — Supplementary file1 (PDF 20795 KB) [file 401_2021_2327_MOESM1_ESM.pdf]

Targeting fibroblast growth factor receptors to combat aggressive ependymoma

Lötsch-Gojo et al.

Online supplementary material

Supplementary Figures 1-18

Supplementary Tables 1-2

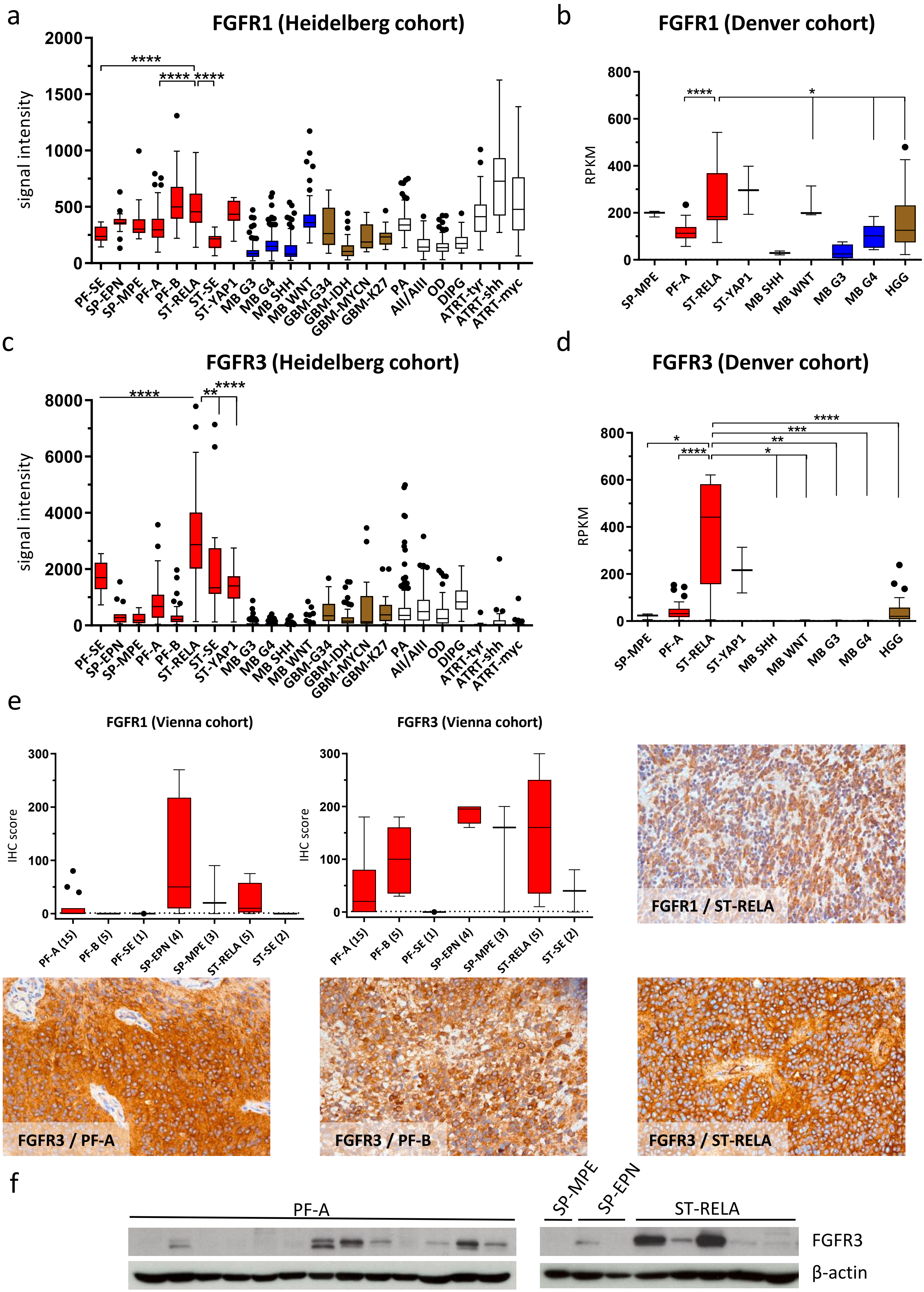

a
FGFR1 (Heidelberg cohort)
signal intensity
PF-SE
SP-EPN
SP-MPE
PF-A
PF-B
ST-RELA
ST-SE
ST-YAP1
MB G3
MB G4
MB SHH
MB WNT
GBM-G34
GBM-IDH
GBM-MYCN
GBM-K27
PA
AII/AIII
OD
DIPG
ATRT-tyr
ATRT-shh
ATRT-myc
b
FGFR1 (Denver cohort)
RPKM
SP-MPE
PF-A
ST-RELA
ST-YAP1
MB SHH
MB WNT
MB G3
MB G4
HGG
c
FGFR3 (Heidelberg cohort)
d
FGFR3 (Denver cohort)
e
FGFR1 (Vienna cohort)
FGFR3 (Vienna cohort)
IHC score
PF-A (15)
PF-B (5)
PF-SE (1)
SP-EPN (4)
SP-MPE (3)
ST-RELA (5)
ST-SE (2)
FGFR1 / ST-RELA
FGFR3 / PF-A
FGFR3 / PF-B
FGFR3 / ST-RELA
f
PF-A
SP-MPE
SP-EPN
ST-RELA
FGFR3
β-actin

Supplementary Figure 1: Validation of FGFR expression by immunohistochemistry (IHC) and in independent datasets. **(a,b,c,d)** FGFR1 and FGFR3 expression levels in Affymetrix gene expression data from the Heidelberg dataset expressed as signal intensity and **(c)** RNA sequencing data from Denver cohort expressed as reads per kilobase million (RPKM) in the indicated samples: ependymoma, medulloblastoma (MB) glioblastoma multiforme (GBM) or high-grade glioma (HGG), pilocytic astrocytoma (PA), astrocytoma (A), oligodendroglioma (OD), diffuse intrinsic pontine glioma (DIPG) and atypical teratoid rhabdoid tumors (ATRT). Significance differences were calculated by one-way ANOVA and with Tukey correction for multiple comparison. **(e)** IHC scores (0-300) of primary ependymomas within the Vienna cohort (upper left panels) as well as represantative images (40x) of FGFR1 and FGFR3 showing cytoplasmic and membranous staining across ependymoma subgroups. **(f)** FGFR3 protein levels determined by Western blot in tissue extracts from posterior fossa group A ependymoma (PF-A), myxopapillary ependymoma (SP-MPE), spinal ependymoma WHO grade II (SP-EPN) and supratentorial ependymoma *RelA* fusion-positive (ST-RELA). **** $p<0.0001$, *** $p<0.001$, ** $p<0.01$, *$p<0.05$

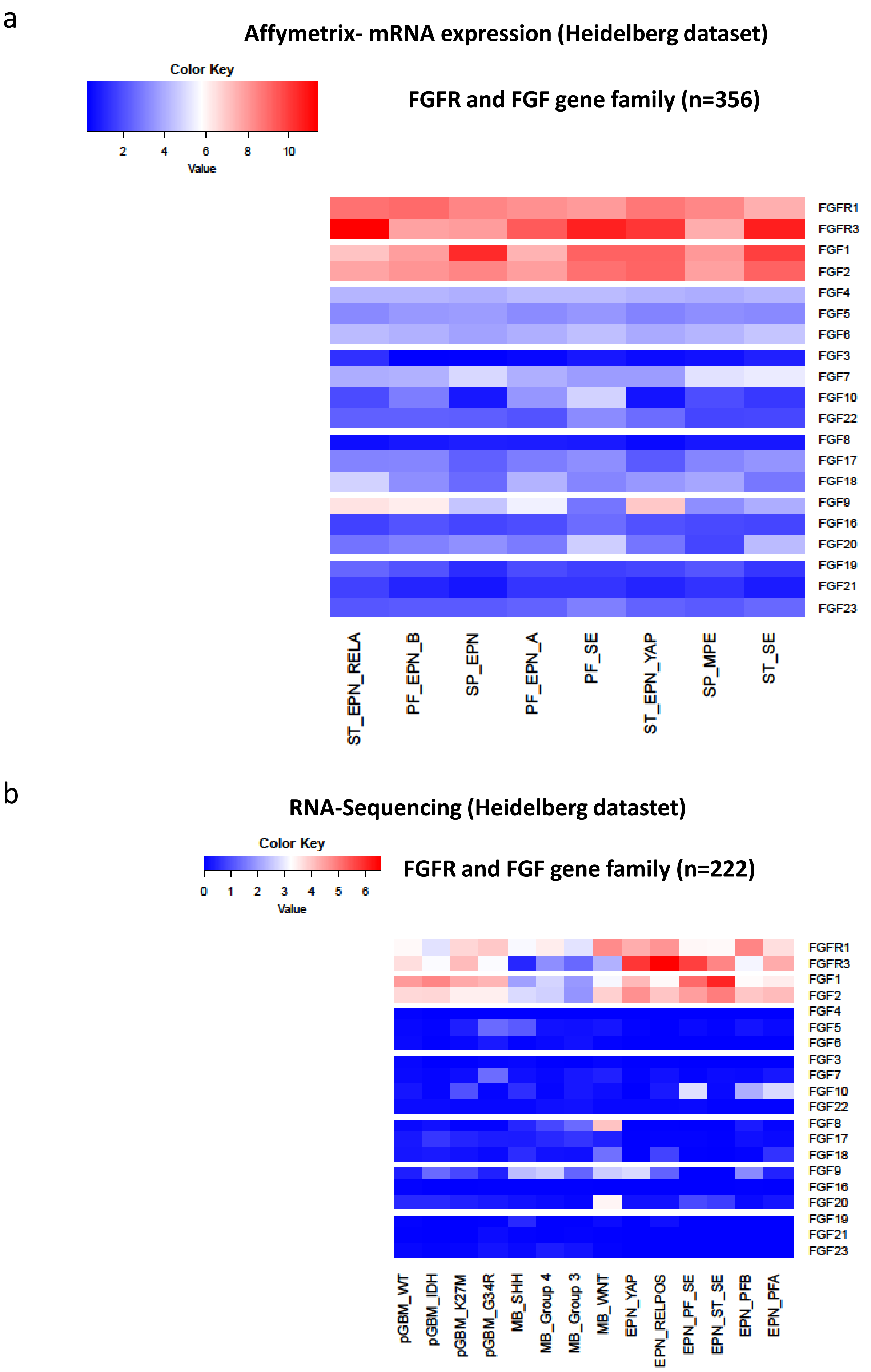
a
Affymetrix- mRNA expression (Heidelberg dataset)
Color Key
2 4 6 8 10
Value
FGFR and FGF gene family (n=356)
FGFR1
FGFR3
FGF1
FGF2
FGF4
FGF5
FGF6
FGF3
FGF7
FGF10
FGF22
FGF8
FGF17
FGF18
FGF9
FGF16
FGF20
FGF19
FGF21
FGF23
ST_EPN_RELA
PF_EPN_B
SP_EPN
PF_EPN_A
PF_SE
ST_EPN_YAP
SP_MPE
ST_SE
b
RNA-Sequencing (Heidelberg datastet)
Color Key
0 1 2 3 4 5 6
Value
FGFR and FGF gene family (n=222)
FGFR1
FGFR3
FGF1
FGF2
FGF4
FGF5
FGF6
FGF3
FGF7
FGF10
FGF22
FGF8
FGF17
FGF18
FGF9
FGF16
FGF20
FGF19
FGF21
FGF23
pGBM_WT
pGBM_IDH
pGBM_K27M
pGBM_G34R
MB_SHH
MB_Group 4
MB_Group 3
MB_WNT
EPN_YAP
EPN_RELPOS
EPN_PF_SE
EPN_ST_SE
EPN_PFB
EPN_PFA

Supplementary Figure 2: Normalized expression levels of FGFR and FGF family genes visualized as heat map. Expression levels are extracted from Affymetrix **(a)** or from RNA sequencing datasets (n=25 EPN, n=167 MB, n=30 GBM) **(b)** derived from the Heidelberg cohort and stratified for the respective EPN subtypes, or compared to high-grade glioma and medulloblastoma. Posterior fossa or supratentorial subependymoma (PF-SE, ST-SE), myxopapillary ependymoma (SP-MPE), spinal ependymoma WHO grade II (SP-EPN), posterior fossa group A (PF-A) and B (PF-B), supratentorial RelA fusion-positive (ST-RELA), and Yap1 fusion-positive (ST-YAP1) ependymomas, medulloblastoma wingless-activated (MB WNT), sonic hedgehog-activated (MB SHH), group 3 (MB G3), and group 4 (MB G4) and glioblastoma multiforme (GBM)

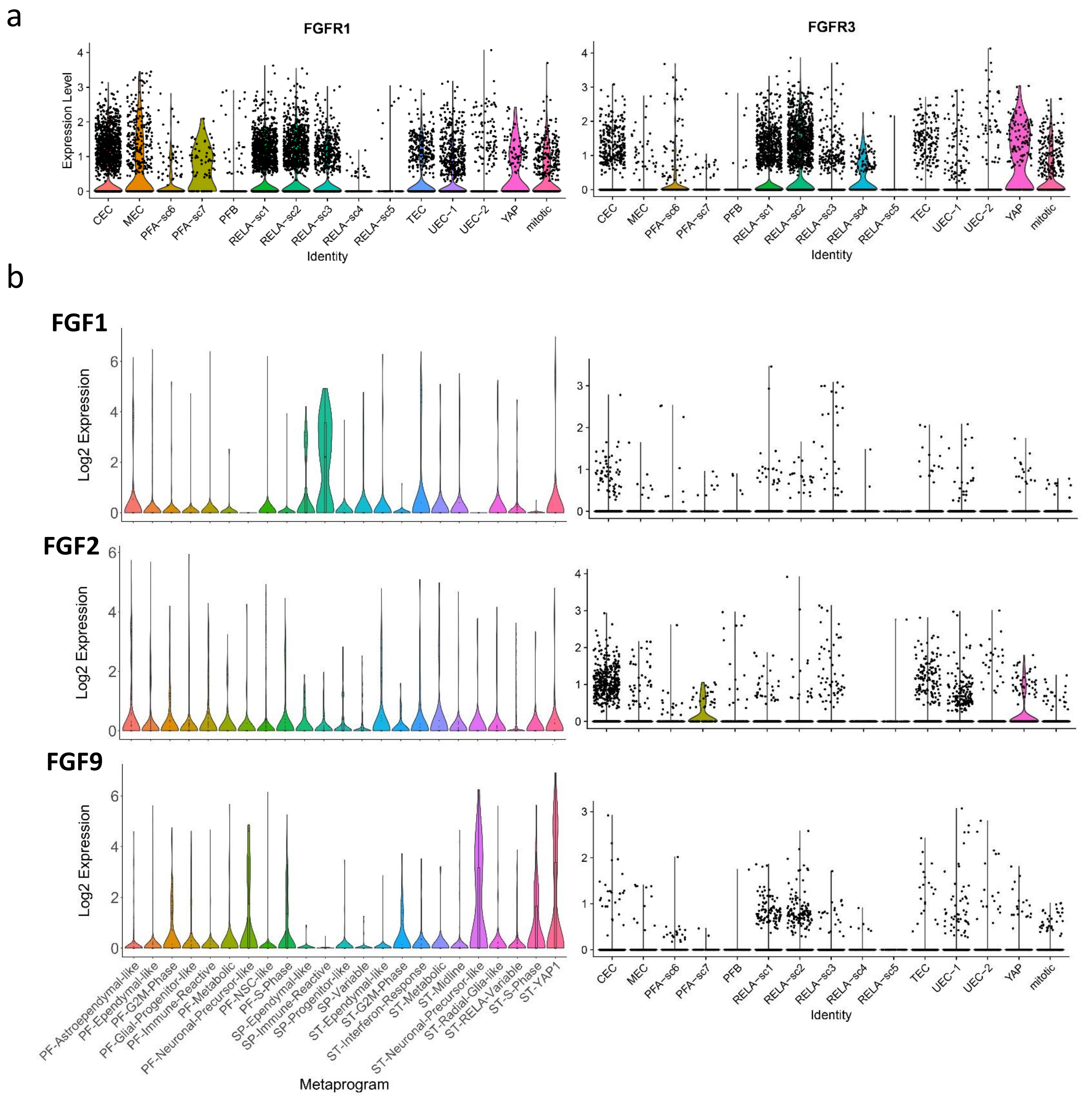

Supplementary Figure 3: *FGF/FGFR* expression in EPN bulk tumor extracts and single cell data. **(a)** Violin plots of *FGFR1* and *FGFR3* expression in EPN cell populations described in Gillen et al. Data were derived from the Pediatric Neuro-oncology Cell Atlas (https://www.pneuroonccellatlas.org/). Black dots indicate outliers. **(b)** Violin plots of *FGF1, FGF2*, and *FGF9* in EPN cell populations described in Gojo et al. (right panels) and Gillen et al. (left panels).

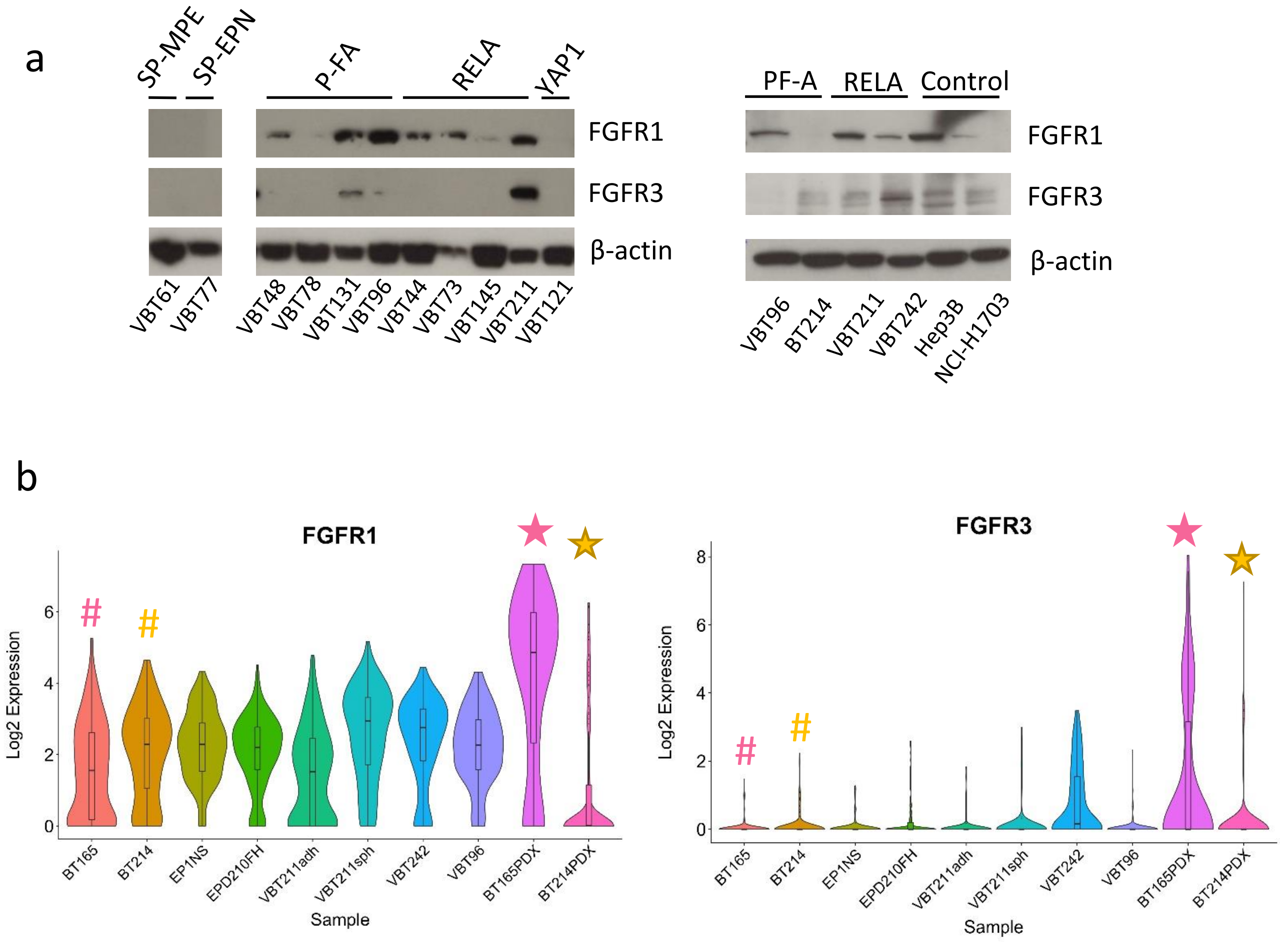

Supplementary Figure 4: *FGFR1* and *FGFR3* expression in EPN cell models. **(a)** Protein expression in cell models (primo-cell cultures and cell lines) of the indicated EPN subgroups analyzed by Western blot. **(b)** Violin plots depict expression levels of *FGFR1* and *FGFR3* determined by scRNA-seq analyses from PF-A and ST-RELA cell models derived directly from tissue extracts or patient-derived xenografts (PDX). Stars highlight PDX models and hashtags the corresponding cell models. adh=adherent, sph=spheres

a

FGFR1

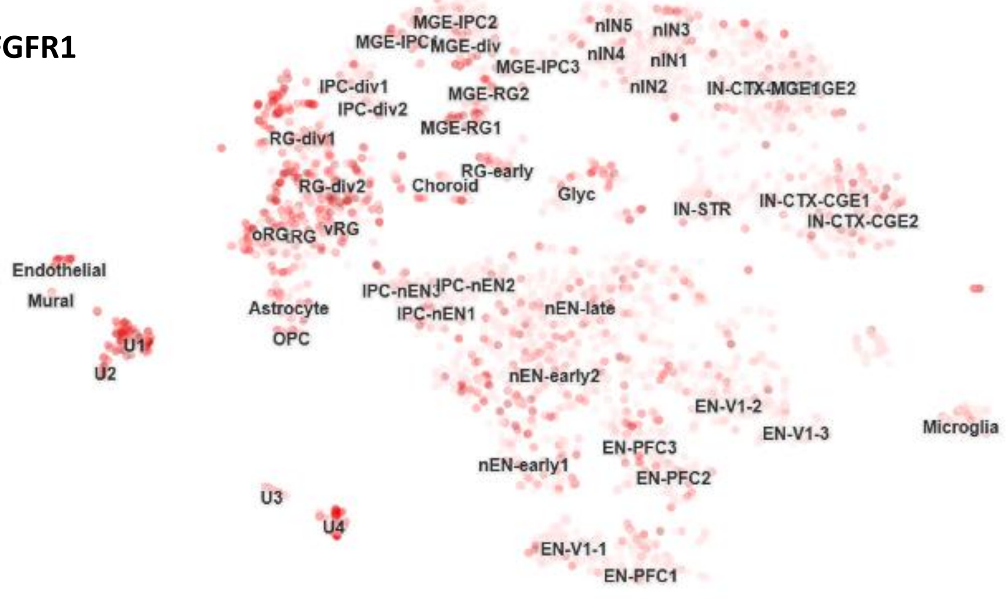

b

FGFR3

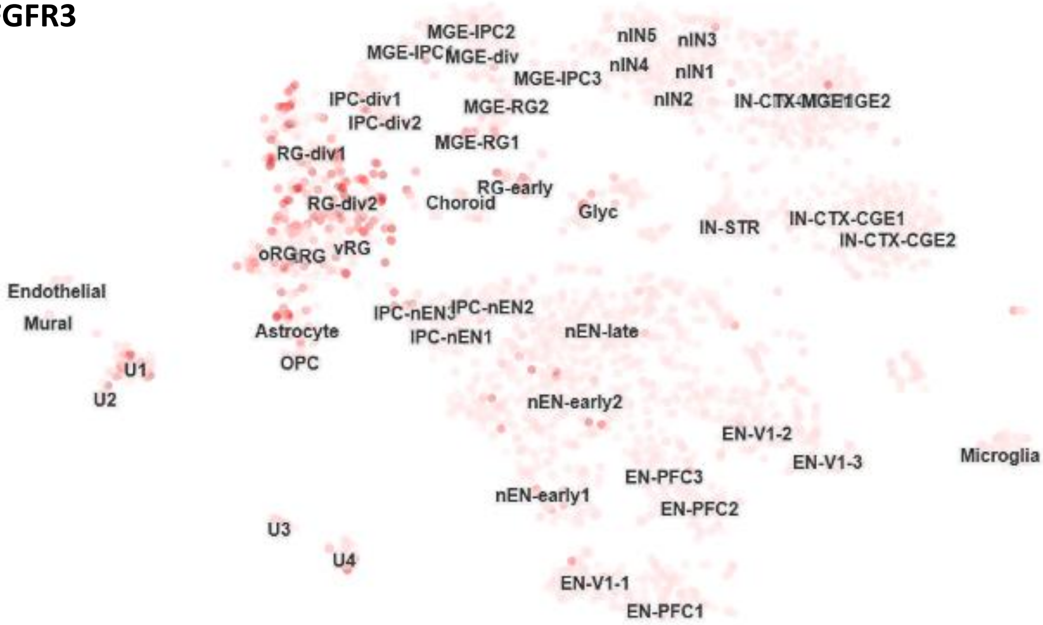

Supplementary Figure 5: *FGFR* expression in cell subpopulations of the human cortex. **(a)** *FGFR1* and **(b)** *FGFR3* expression in scRNA-seq data of the human cortex 34. Data was derived and analyzed with UCSC cell browser (https://cells.ucsc.edu/). Red indicates higher cellular expression levels. Nomenclature of cell populations is depicted as published 34. Most important populations with respect to *FGFR* expression include RG (radial glia cells) and U1/2 (unknown population 1/2).

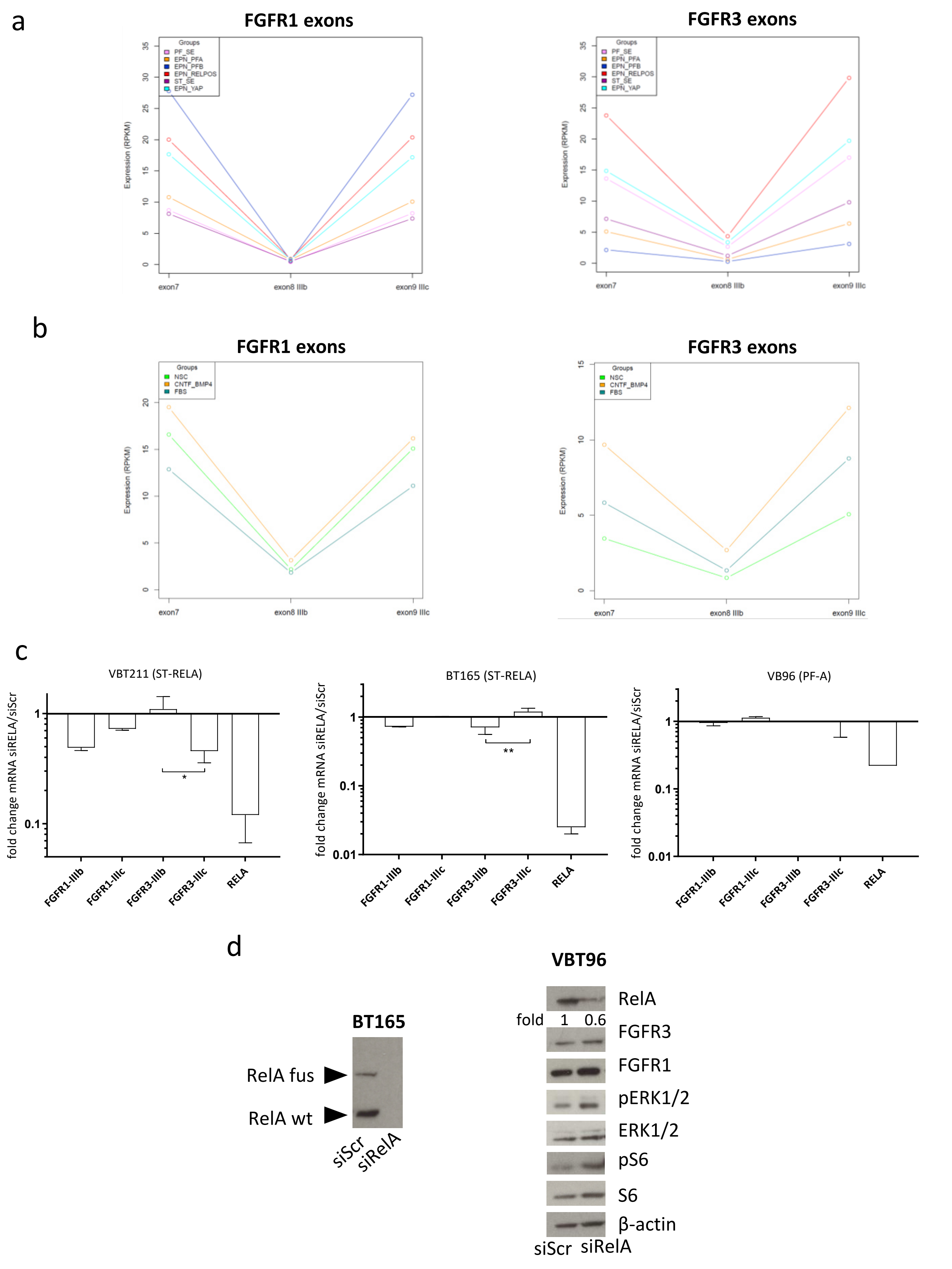

a
FGFR1 exons
FGFR3 exons
Groups
PF_SE
EPN_PFA
EPN_PFB
EPN_RELPOS
ST_SE
EPN_YAP
Expression (RPKM)
exon7
exon8 IIIb
exon9 IIIc
b
FGFR1 exons
FGFR3 exons
Groups
NSC
CNTF_BMP4
FBS
c
VBT211 (ST-RELA)
BT165 (ST-RELA)
VB96 (PF-A)
fold change mRNA siRELA/siScr
FGFR1-IIIb
FGFR1-IIIc
FGFR3-IIIb
FGFR3-IIIc
RELA
d
BT165
RelA fus
RelA wt
siScr
siRelA
VBT96
RelA
fold 1 0.6
FGFR3
FGFR1
pERK1/2
ERK1/2
pS6
S6
β-actin
siScr siRelA

Supplementary Figure 6: FGFR1 and FGFR3 splicing and regulation. **(a)** Expression levels of exon 7 (both variants), exon 8 (IIIb-specific), and exon 9 (IIIc-specific) within the Heidelberg RNA-seq datasets, expressed as RPKM values in the indicated EPN subtypes. **(b)** Expression levels of exon 7 (both variants), exon 8 (IIIb-specific), and exon 9 (IIIc-specific) within human neural stem cell (NSC) cultures and derived astrocytic cell cultures in either FBS (fetal bovine serum) or CTNF/BMP4 containing media derived from GSE76122. **(c)** mRNA expression levels 72h post transfection of ependymoma cell models with siRELA or non-targeting siRNA (siScr), depicted as bar graphs. Fold changes of the indicated genes (normalized to the housekeeping gene β-actin) are given relative to respective siScr controls. Data are presented as mean ± SD of two independent experiments performed in triplicates. Significant differences between IIIb and IIIc variants were analyzed by two-tailed Student's t-test. **(d)** Protein expression and phosphorylation levels 72h post transfection with siRELA or non-targeting siRNA (siScr) of indicated ependymoma cell models depicted as immunoblots. The left panel indicates the ZFTA-RELA fusion protein (fus) and RELA wild-type (wt) bands. Fold changes of the indicated proteins (normalized to the housekeeping gene β-actin) are given relative to respective siScr controls. $^{*}p < 0.05$

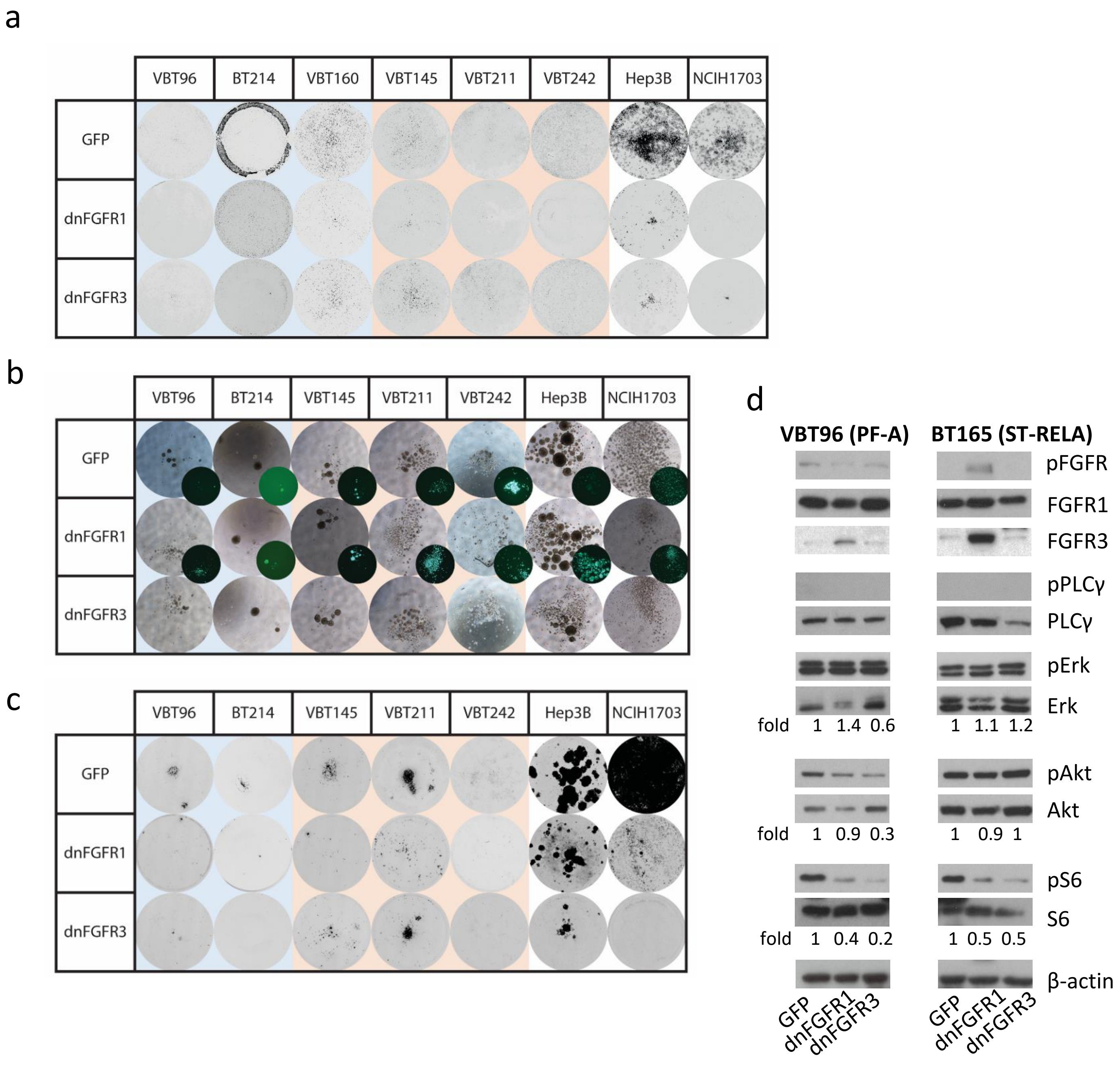

Supplementary Figure 7: Expression of dominant-negative (dn) FGFR1 and FGFR3 impacts clone formation and stem cell characteristics of EPN cells. **(a)** Representative photographs of clonogenic cell growth upon introduction of an adenovirus encoding empty vector (GFP), dnFGFR1 or dnFGFR3 in the indicated PF-A (blue) and ST-RELA (pink) EPN cell models. **(b)** Spheroids grown in stem-cell medium are shown three days after infection with the indicated adenoviral constructs in different EPN PF-A (blue) and ST-RELA (pink) cells. Green fluorescent pictures confirm infection efficacy of cells expressing empty vector or dnFGFR1. **(c)** Stem cell plasticity of manipulated spheres was investigated by testing the re-growth in medium containing 10% FCS. Representative pictures of attached spheres from EPN cell models and FGFR-driven controls are depicted. In all panels, Hep3B and NCI-H1703 served as positive controls for FGFR3 and FGFR1, respectively. **(d)** Western blot analyses of the PF-A (VBT96) and ST-RELA (BT165), cell models upon expression of dnFGFR1 and dnFGFR3. Total protein expression and phosphorylation levels of the indicated FGFRs (pFGFR, FGFR1/3), PLC-gamma (PLCγ, pPLCγ), MAPK (ERK, pERK) and PI3K (Akt, pAkt, S6, pS6) pathway mediators are depicted. ß-actin served as loading control. Fold changes of the indicated proteins are given relative to respective GFP-transduced controls., GFP=green fluorescent protein

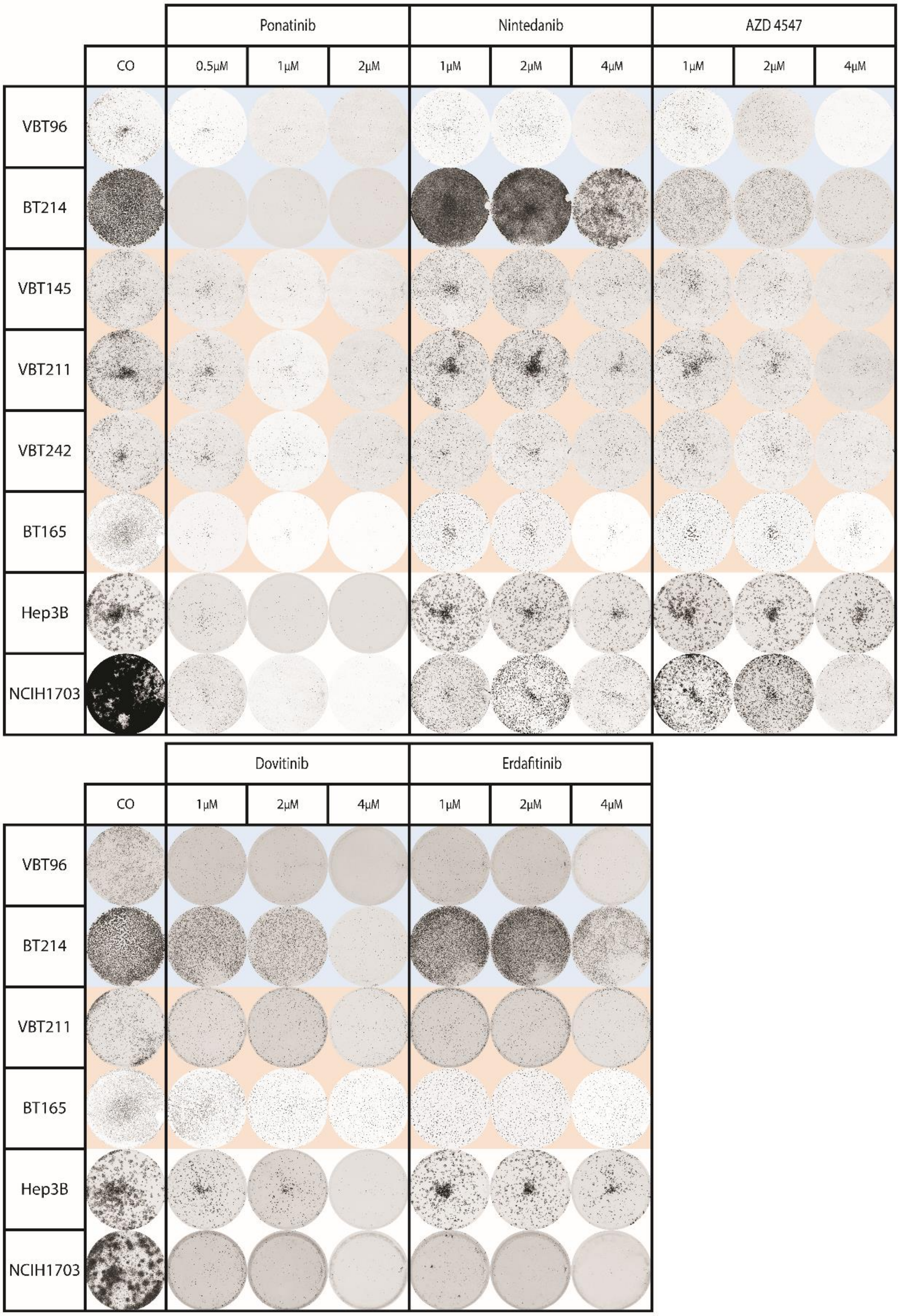

Supplementary Figure 8: Exposure of EPN cells to FGFRis impacts on clonogenic survival. Representative pictures of clonogenic cell growth upon treatment with ponatinib, nintedanib and AZD-4547 (upper panels) as well as with dovitinib and erdafitinib (lower panels) in the indicated PF-A (blue) and ST-RELA (pink) EPN cell models.

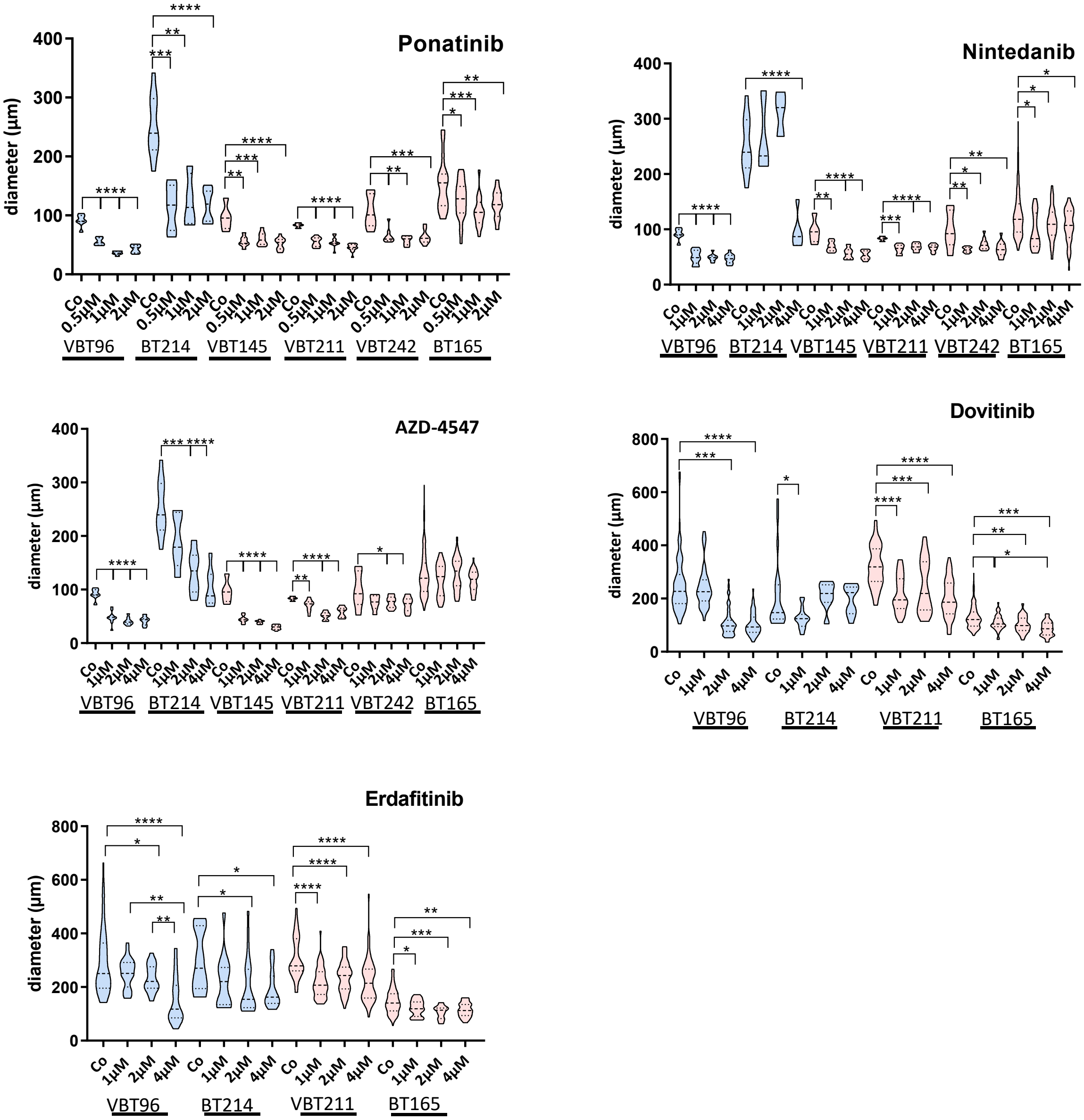

Supplementary Figure 9: Treatment with FGFRis reduces three-dimensional spheroid growth. Sphere diameters (in µm) of PF-A (blue) and ST-RELA (pink) cell models upon treatment with ponatinib, nintedanib, AZD-4547, dovitinib and erdafitinib are shown as violin blots. Statistical differences between untreated and drug-exposed samples were determined by one-way ANOVA with Tukey correction for multiple comparison. **** $p<0.0001$, *** $p<0.001$, ** $p<0.01$, * $p<0.05$

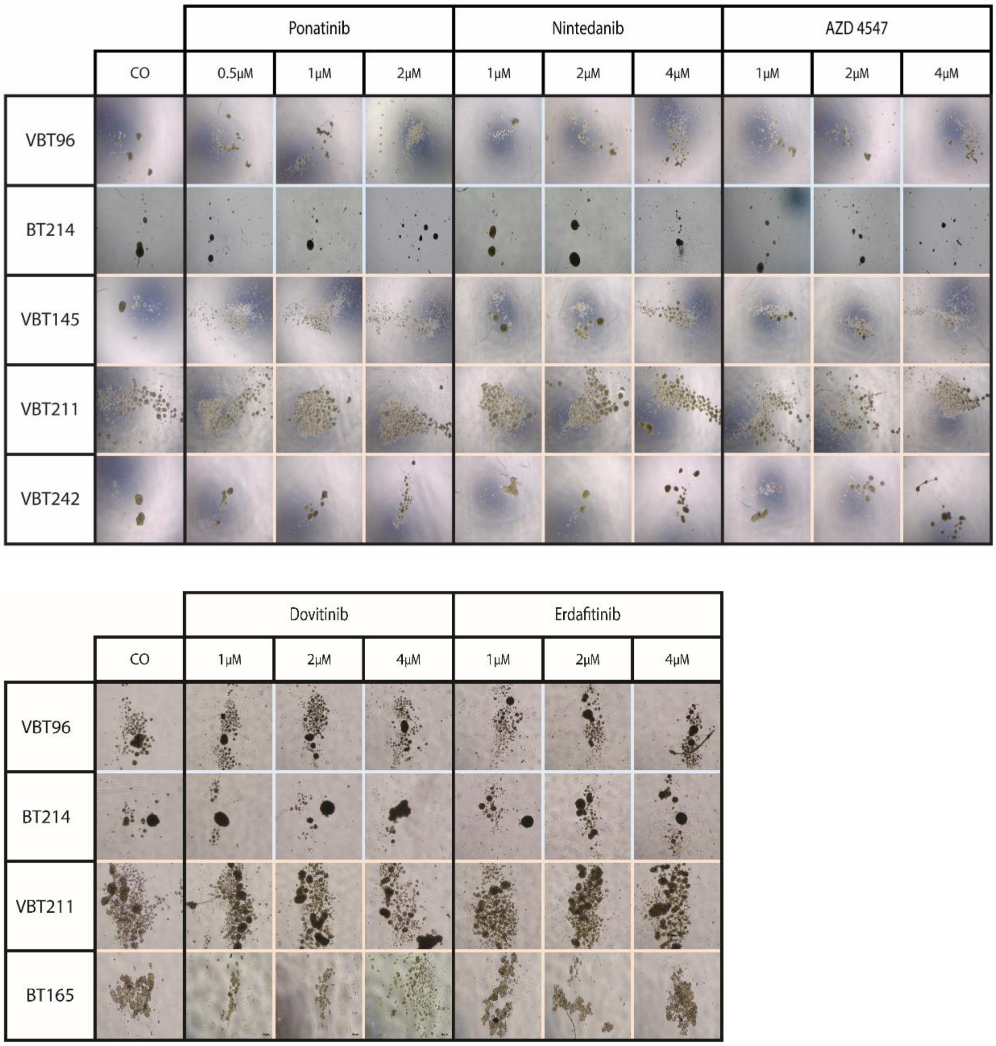

Supplementary Figure 10: Exposure of EPN cell models to FGFRis impacts on stem cell characteristics. Spheroids are shown three days after treatment with the indicated inhibitors in different EPN PF-A (blue) and ST-RELA (pink) cells.

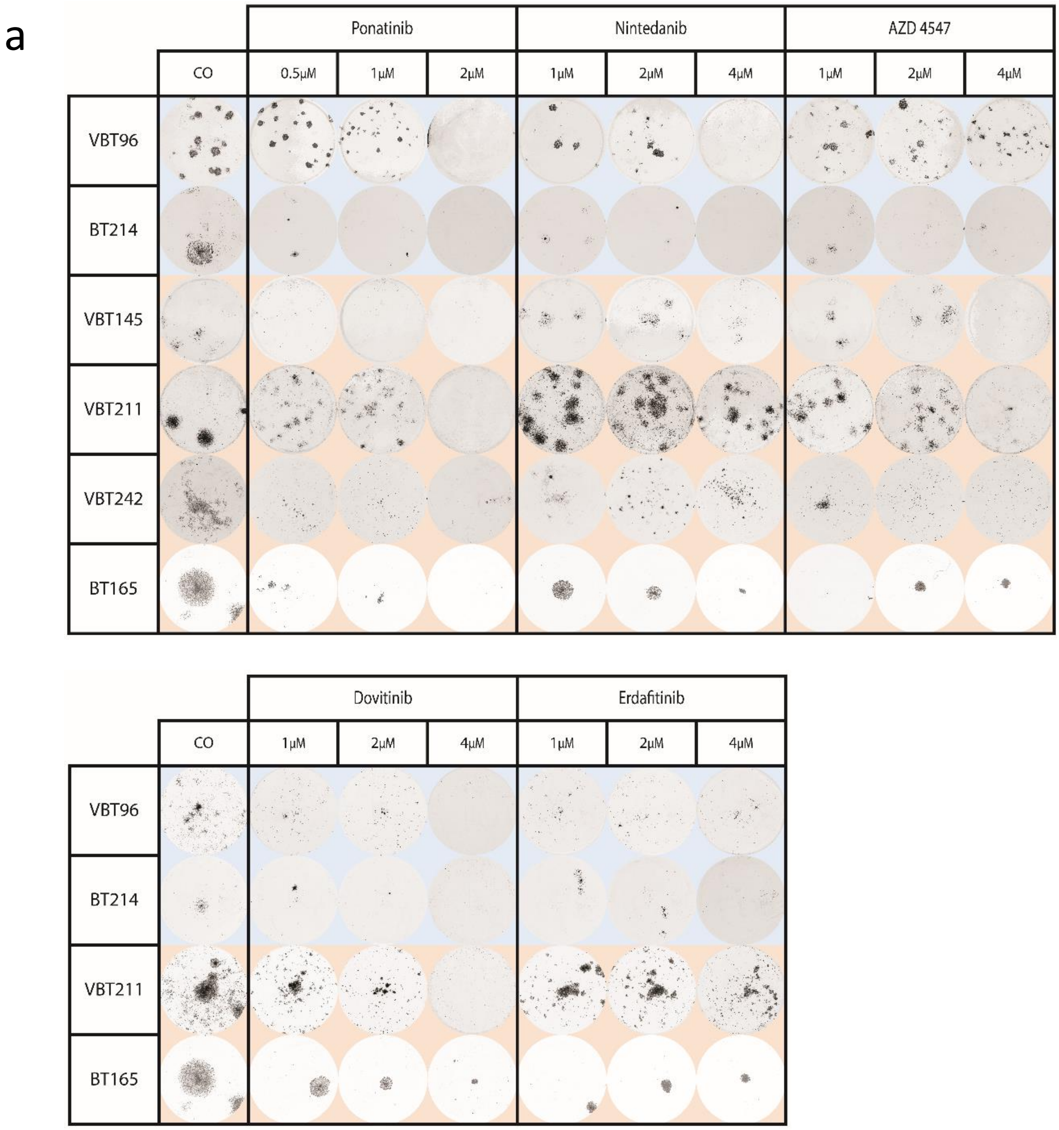

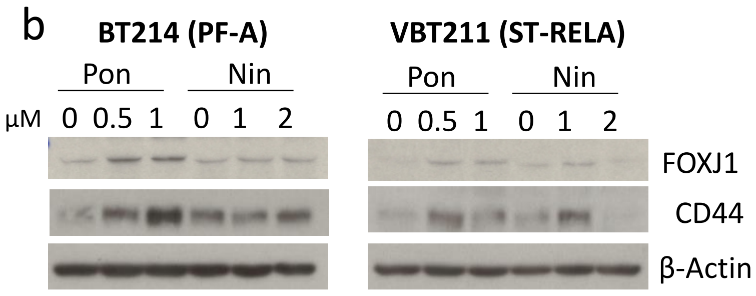

Supplementary Figure 11: Exposure of EPN cell models to FGFRis impacts on stem cell characteristics.

(a) Re-attachment/differentiation of treated spheres was investigated by testing the re-growth in medium containing 10% FCS. Representative pictures of attached spheres from EPN cell models are depicted. **(b)** Stem cell/differentiation markers (CD44 and FOXJ1) in PF-A (BT214) and ST-RELA (VBT211) cells upon treatment with ponatinib (Pon) and nintedanib (Nin) with the indicated concentrations for 24 hours was analyzed by Western blot. ß-actin served as loading control.

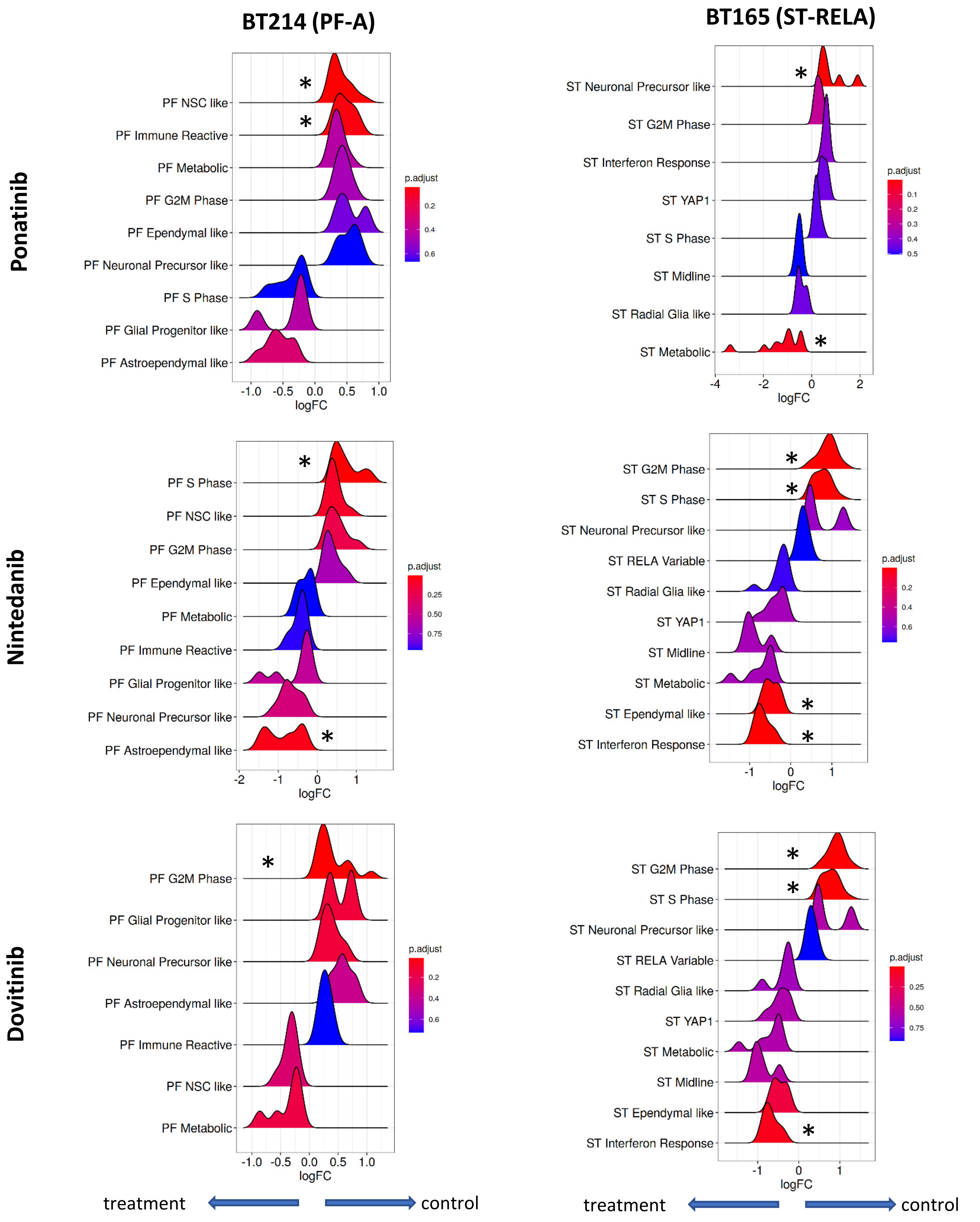

Supplementary Figure 12: Ridge plots derived from gene set enrichment analyses with the indicated ependymoma gene signatures. Positive values indicate enrichment in control, whereas negative values indicate enrichment in treated cells. Original data are summarized in supplementary file 2. *, p<0.05

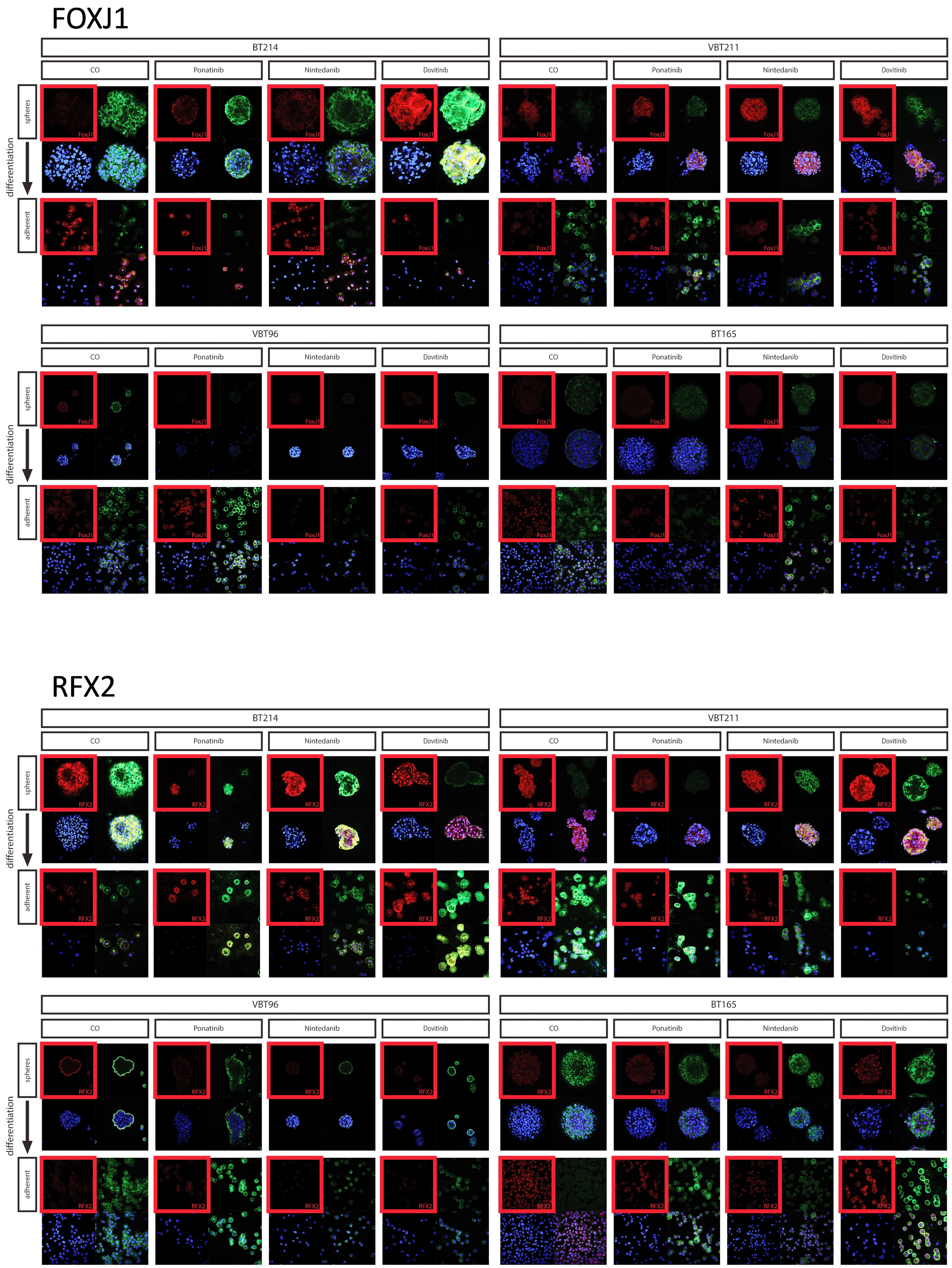

Supplementary Figure 13: Immunofluorescence staining of FOXJ1 and RFX2 in ependymoma spheres and upon redifferentiation as indicated. Spheres were treated with 0.5µM ponatinib, 2µM nintedanib or 2µM dovitinib for 6 days (upper panels) followed by reseeding into FCS-containing media and culture for 3 days (lower panels).

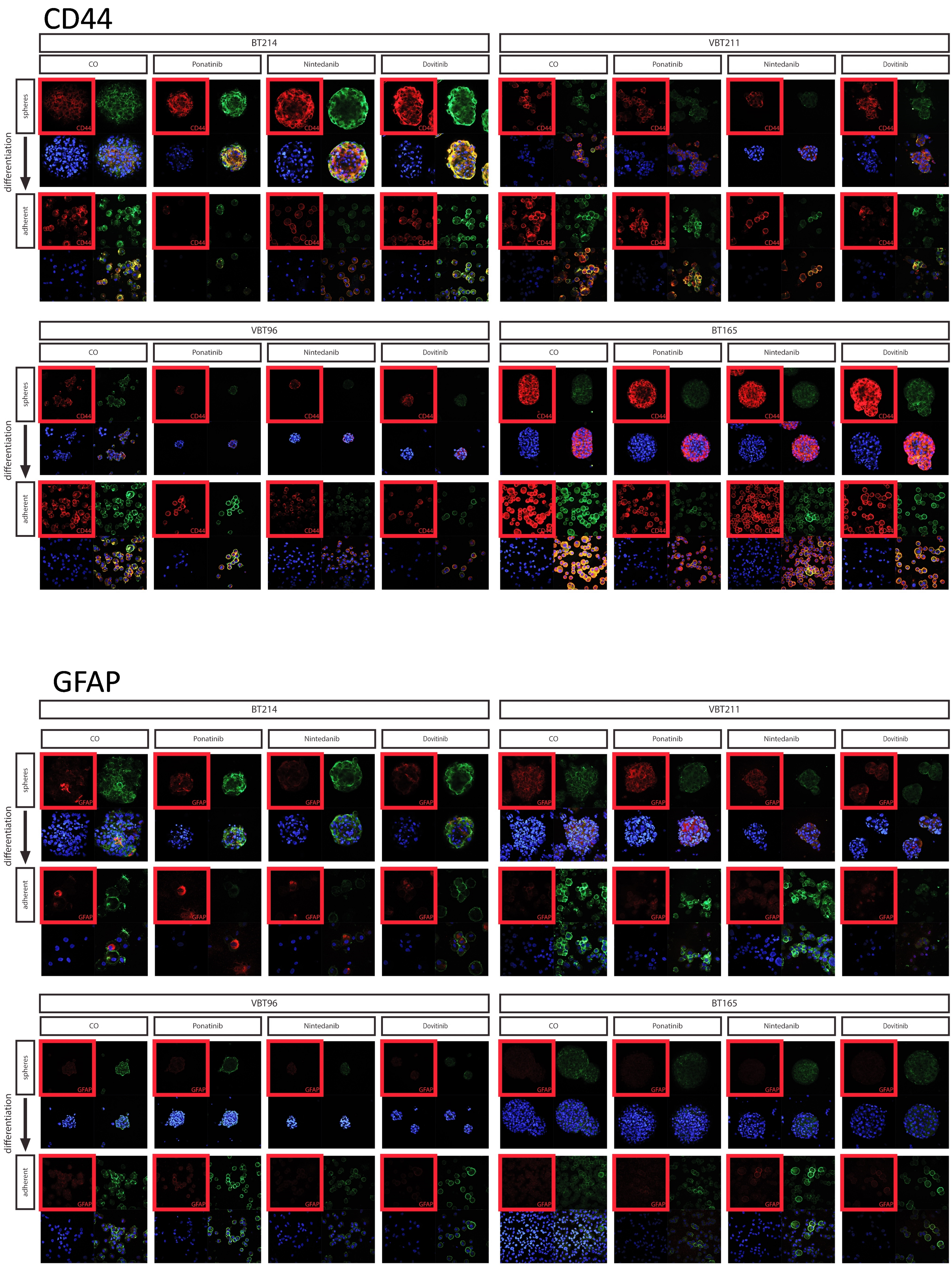

Supplementary Figure 14: Immunofluorescence staining of CD44 and GFAP in ependymoma spheres and upon redifferentiation as indicated. Spheres were treated with 0.5µM ponatinib, 2µM nintedanib or 2µM dovitinib for 6 days (upper panels) followed by reseeding into FCS-containing media and culture for 3 days (lower panels).

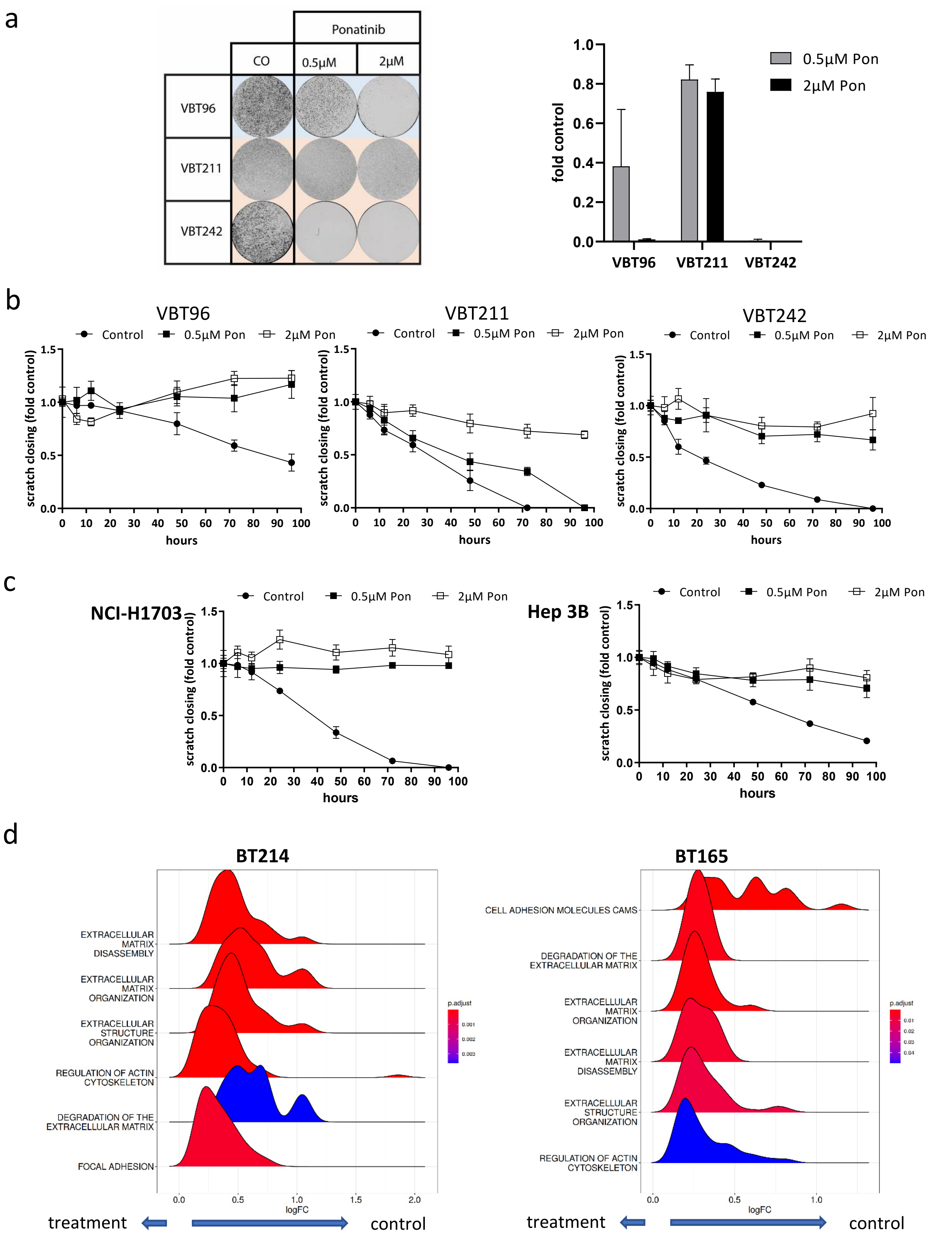
a
Ponatinib
CO
0.5µM
2µM
VBT96
VBT211
VBT242
fold control
0.5µM Pon
2µM Pon
b
VBT96
VBT211
VBT242
Control
0.5µM Pon
2µM Pon
scratch closing (fold control)
hours
c
NCI-H1703
Hep 3B
Control
0.5µM Pon
2µM Pon
scratch closing (fold control)
hours
d
BT214
BT165
EXTRACELLULAR MATRIX DISASSEMBLY
EXTRACELLULAR MATRIX ORGANIZATION
EXTRACELLULAR STRUCTURE ORGANIZATION
REGULATION OF ACTIN CYTOSKELETON
DEGRADATION OF THE EXTRACELLULAR MATRIX
FOCAL ADHESION
CELL ADHESION MOLECULES CAMS
p.adjust
logFC
treatment
control

Supplementary Figure 15: Ponatinib treatment reduced the migratory potential of EPN cells. **(a)** Effects of ponatinib (Pon, 0.5 and 2µM) inhibition on cell migration were determined by transwell-migration assays. Cells were seeded in transwell cell culture inserts in serum-free growth media. The ability of the cells to migrate through a porous filter attracted by serum gradient was tested after 18h (VBT211) or 72h (VBT96 and VBT242). Representative photographs of cells at the bottom of filters stained with crystal violet are shown (left panel). The respective quantification, giving the fold changed migratory potential in comparison to untreated controls is depicted (right panel). Bars represent the means ± SD of two independent experiments performed in duplicates. **(b and c)** Wound healing assays were analyzed by live-cell-microscopy in PF-A (VBT96) and ST-RELA (VBT211 and VBT242) cell models upon exposure to ponatinib at the indicated concentration (b) and compared to FGFR-positive controls, NCI-H1703 and Hep3B (c). Quantification of scratch width is depicted as means ± SD of each time point investigated. **(d)** Ridge plots derived from gene set enrichment analyses with the indicated migration-related gene sets. Arrows indicate enrichment in the respective control or treated ependymoma cells. Original data are summarized in supplementary file 2. *, $p<0.05$

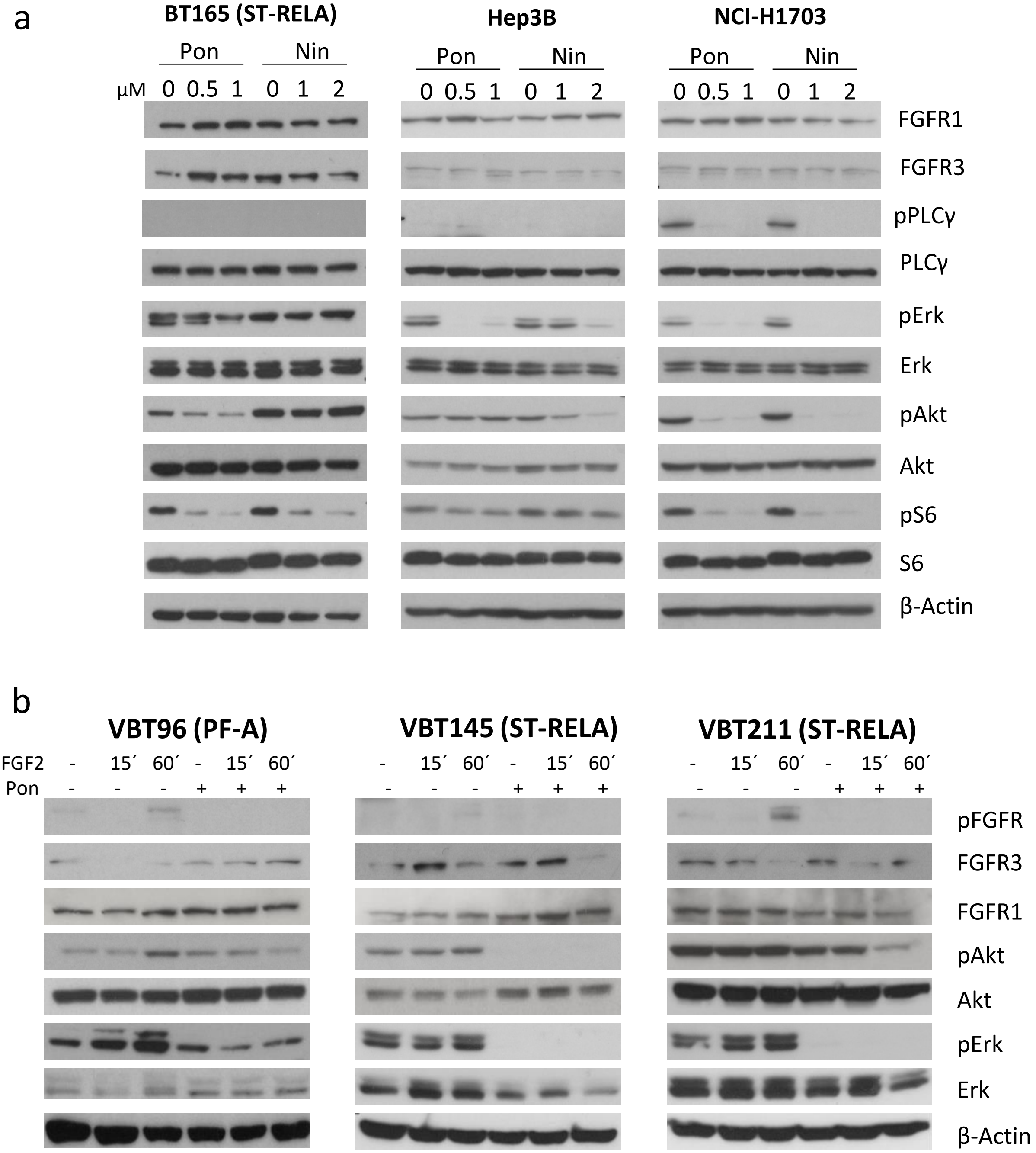

Supplementary Figure 16: Effects of FGFR inhibition on FGFR downstream signaling cascades in FGFR-driven control cells and on patient treatment. (a) Effects of long-term treatment (24h) with the indicated concentrations of ponatinib (Pon) and nintedanib (Nin) on FGFR1 and FGFR3, as well as on PLCγ, MAPK and PI3K signaling activation (indicated by changes in the phosphorylation status) in BT165, Hep3B and NCI-H1703 cells was analyzed by Western blot. **(b)** Effects on FGFRs, MAPK, and PI3K signaling activation upon stimulation of starved EPN cell models with FGF2 (20ng/ml) at indicated time points and additional ponatinib (5μM) treatment (4 hours preincubation). ß-actin served as loading control.

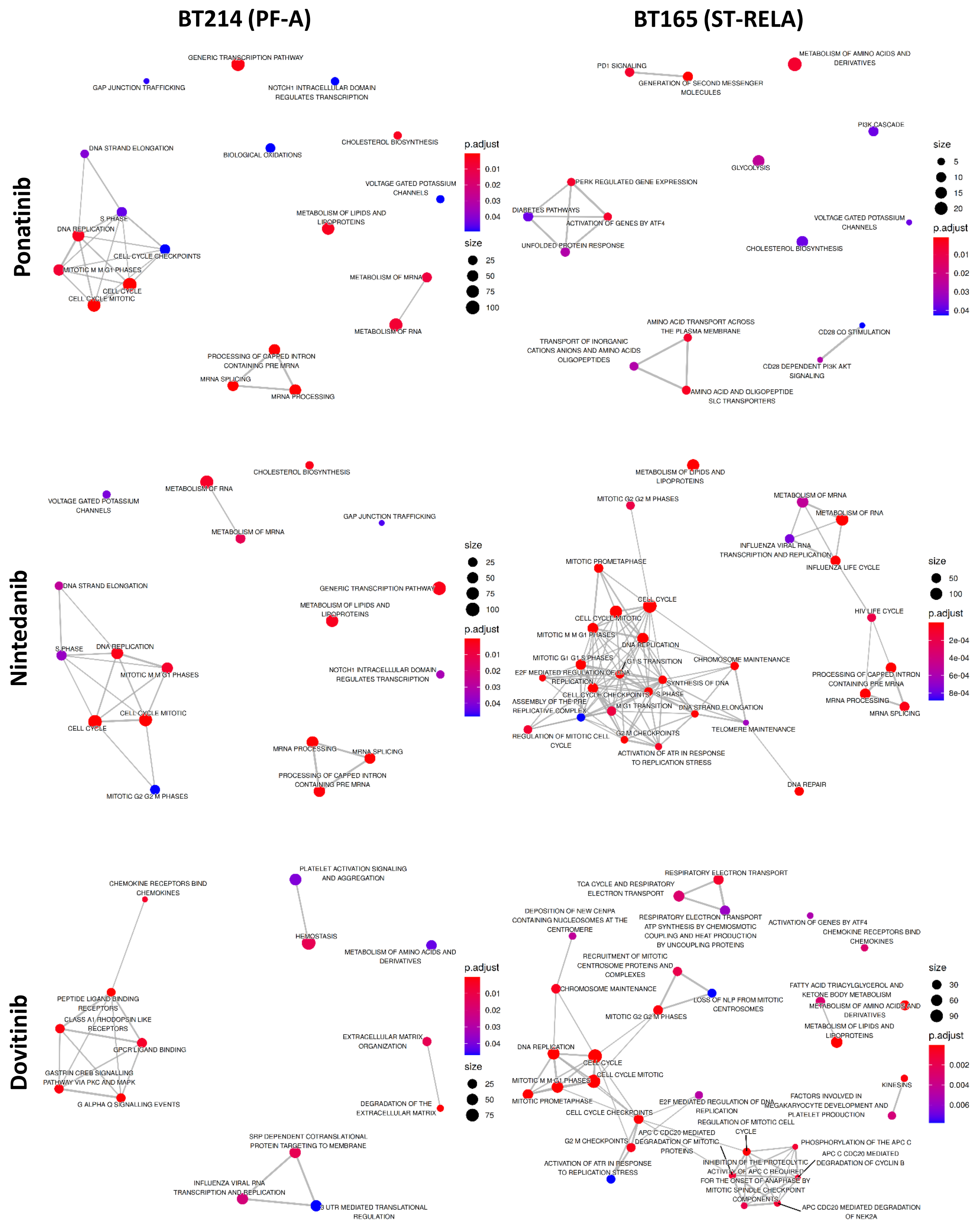

Supplementary Figure 17: Enrichment maps of REACTOME gene sets (top 30 enriched gene sets, p<0.05). Original data are summarized in supplementary file 2.

after resection

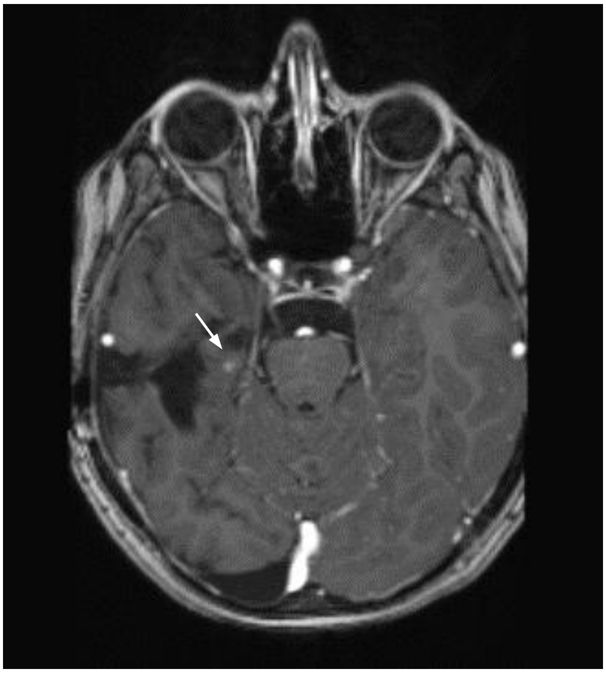

Supplementary Figure 18: Axial contrast-enhanced T1-weighted magnetic resonance images (MRI) showing periventricular residual tumor which could not be resected.

Table S1.: Primary cell models and stable cell lines investigated in course of this study

	Molecular Subgroup	Primary/recurrence	Stable cell line	CNVs
VBT77	SP-EPN	Recurrence		n.a.
VBT78	PF-EPN-A	Recurrence		balanced
VBT96	PF-EPN-A	Recurrence	x	1q / 4 / 5 gain, Chr 6 loss
VBT131	PF-EPN-A	Recurrence		Chr6/7q/10 p loss
VBT160	PF-EPN-A	Recurrence		n.a.
BT214	PF-EPN-A	Recurrence	X	balanced
VBT73	ST-EPN-RELA	Recurrence	x	CDKN2A / 22p del.
VBT145	ST-EPN-RELA	Recurrence	x	CDKN2A / 22p del.
VBT211	ST-EPN-RELA	Recurrence	x	CDKN2A / 2q / 14q / 22p del.
VBT242	ST-EPN-RELA	Recurrence	x	1q gain, CDKN2A del.
VBT371	ST-EPN-RELA	Recurrence	x	CDKN2A / 2q / 14q / 22p del.
BT165	ST-EPN-RELA	not known	X	CDKN1A del.

Table S2: List of primary and secondary antibodies

Antibody	Company	WB	IF
Primary			
PLC-gamma-1 (D9H10)	Santa Cruz Biotechnology (Dallas, Texas, USA)	X	
phospho-PLC-gamma-1 (Tyr783)	Cell Signaling Technology	X	
FGFR-1 (D8E4) XP	Cell Signaling Technology	X	
phospho-FGFR (Tyr653/654)	Cell Signaling Technology	X	
FGFR-3 (E-7)<br>detecting: non-glycosylated FGFR-3: 97 kDa, FGFR-3 precursor: 125 kDa<br>mature FGFR-3: 135 kDa.	Santa Cruz Biotechnology	X	
ERK1/2; p44/42	Cell Signaling Technology	X	
phospho-ERK1/2; p-p44/42 (Thr202/Tyr204)	Cell Signaling Technology	X	
Akt (pan) (C67E7)	Cell Signaling Technology	X	
phospho-Akt (Ser473) (D9E)	Cell Signaling Technology	X	
S6; Ribosomal Protein (C-8)	Santa Cruz Biotechnology	X	
Phospho-S6; Ribosomal Protein (Ser 240/244)	Cell Signaling Technology	X	
β-actin (A5441)	Sigma-Aldrich	X	X
β-actin (D6A8)	Cell Signaling Technology		X
FOXJ1 (3-19)	Santa Cruz Biotechnology	X	X
CD44 (156-3C11)	Cell Signaling Technology		X
GFAP (G-A-5)	Sigma-Aldrich		X
RFX2 (PA5-61850)	Thermo Fisher Scientific		X
Secondary			
mouse-anti-rabbit IgG-HRP (sc-2357)	Santa Cruz Biotechnology	X	
mouse IgG-HRP (GTX213111-01)	GeneTex (Irvine, California, USA)	X	
goat anti-mouse IgG, Alexa Fluor 633 (A-21050)	Thermo Fisher Scientific		X
goat anti-mouse IgG, Alexa Fluor 594 (A-11032)	Thermo Fisher Scientific		X
goat anti-rabbit, Alexa Fluor 633 (A-21070)	Thermo Fisher Scientific		X
goat anti-Rabbit IgG, Alexa Fluor 594 (A-11012)	Thermo Fisher Scientific		X
